# Sequential CRISPR-EspCas9-Mediated Wild-Type Depletion Enhances the Detection Sensitivity of Rare Mutations for Canine Liquid Biopsy Application

**DOI:** 10.3390/bios16060330

**Published:** 2026-06-10

**Authors:** Sumin Hong, Chul-Sung Park, Kyung Wook Been, Seunghun Kang, Jaewoo Hong, Jung-whan Kim, Junho K. Hur

**Affiliations:** 1 Graduate School of Biomedical Science and Engineering, Hanyang University, Seoul 04763, Republic of Korea; sumin1716@naver.com; 2 Hanyang Biomedical Research Institute, Hanyang University, Seoul 04763, Republic of Korea; chulsung12@hanyang.ac.kr (C.-S.P.); ysbkw@hanyang.ac.kr (K.W.B.); 3 Life Science Research Institute, Korea Advanced Institute of Science and Technology (KAIST), Daejeon 34141, Republic of Korea; rkdekffyd@naver.com; 4 Department of Physiology, Daegu Catholic University School of Medicine, Duryugongwon-ro 17-gil 33, Nam-gu, Daegu 42472, Republic of Korea; 5 CaniCatiCare, Inc., 15 Dalgubeoldaero 528-gil, Suseong-gu, Daegu 42078, Republic of Korea; 6 Summerville Pet Clinic, Summerville, SC 29485, USA; 7 Department of Genetics, College of Medicine, Hanyang University, Seoul 04763, Republic of Korea; 8 Hanyang Institute of Bioscience and Biotechnology, Hanyang University, Seoul 04763, Republic of Korea

**Keywords:** canine cancer, cell-free DNA, CRISPR-EspCas9, liquid biopsy, mutation detection, *PIK3CA*, sequential enrichment, veterinary oncology

## Abstract

One of the major obstacles in early cancer detection in dogs is the limited sensitivity in detecting circulating tumor DNAs (ctDNAs) with low abundances. Standard next-generation sequencing (NGS) without error correction typically achieves detection limits around ~1% mutant allele frequency (MAF). We sought to improve the detection sensitivity using a sequential CRISPR-EspCas9 enrichment strategy in which iterative in vitro cleavage (IVC) was combined with PCR amplification to selectively deplete wild-type DNA and enrich rare tumor mutations. Applying the strategy to genomic DNA and cell-free DNA mimics from canine mammary gland tumor cell lines demonstrated that IVC enrichment enabled the detection of cancer-associated *PIK3CA* H1047R mutations that were undetectable by conventional Sanger sequencing. To evaluate detection sensitivity, we characterized enrichment using synthetic templates for *PIK3CA* H1047R and other cancer-related mutations, *BRAF* V596E, and *KRAS* G12C. We observed that three iterations of sequential IVC achieved ~160, ~15, and ~2.2-fold enrichment for *PIK3CA* H1047R, *BRAF* V596E, and *KRAS* G12C, respectively. Under the present synthetic-template conditions, the analytical LOD reached 0.001% MAF for *PIK3CA* and 0.01% MAF for *BRAF*, whereas *KRAS* showed only modest enrichment and remained practically limited under the current guide design. Together, the results show that the CRISPR-EspCas9 IVC strategy enables selective enrichment of low-frequency single-nucleotide mutant alleles. We anticipate that the finding could be utilized to develop a highly sensitive veterinary liquid biopsy application with further optimization and validation using canine plasma cfDNA.

## 1. Introduction

Cancer is one of the leading causes of mortality in older dogs, affecting approximately 50% of animals over 10 years of age [1,2]. The emotional and economic burden on pet owners is substantial, with treatment costs frequently exceeding thousands of dollars and often yielding limited success when tumors are detected at advanced stages [3]. Current diagnostic approaches—including physical examination, imaging, and tissue biopsy—typically identify cancers only after clinical manifestation, when therapeutic options are constrained, and prognoses are often poor [4,5]. This diagnostic delay underscores an urgent need for early, non-invasive detection methods capable of identifying cancers at early treatable stages.

Circulating cell-free DNA (cfDNA) has emerged as a promising biomarker source for minimally invasive cancer detection [6]. Tumor cells release DNA fragments into the bloodstream through apoptosis, necrosis, and active secretion, providing a “liquid biopsy” that can be obtained through simple blood collection [7]. This approach is minimally invasive, repeatable, and amenable to real-time monitoring of disease progression and treatment responses [8,9]. In veterinary oncology, canine liquid biopsy generally follows the same broad principle as human liquid biopsy, namely the analysis of blood-derived cfDNA or ctDNA. Recent studies have reported NGS-based cfDNA profiling for non-invasive cancer detection and monitoring in dogs, and ddPCR-based detection of *PIK3CA* H1047R in plasma and serum from dogs with canine mammary tumors was demonstrated [10,11,12]. Moreover, cfDNA concentration or fragmentation analysis was applied as a detection method for potential minimally invasive biomarkers in canine mammary tumors. These studies support the feasibility of canine liquid biopsy, while also suggesting that veterinary liquid biopsy may be less standardized and could benefit from more sensitive pre-analytical enrichment strategies.

However, a critical limitation impedes the clinical utility of cfDNA-based cancer detection. Circulating tumor DNA (ctDNA) carrying cancer-specific mutations typically represents less than 0.1%—and frequently less than 0.01%—of total cfDNA, particularly in early-stage diseases [13,14]. The low mutant allele frequencies (MAF) pose a formidable detection challenge: direct Sanger sequencing achieves detection limits of only 10–20%, and the sensitivity of next-generation sequencing (NGS) without error correction is typically limited to approximately 1% [15,16]. Thus, a substantial gap exists between the clinical need for early detection at sub-0.1% frequencies and the current technical capabilities.

Canine mammary tumors represent one of the most common neoplasms in intact female dogs, and *PIK3CA*, *BRAF*, and *KRAS* genes frequently acquire mutations in these malignancies [17,18]. Whole-exome sequencing studies have identified *PIK3CA* as the most frequently mutated gene in canine mammary tumors, with the H1047R hotspot mutation occurring in 25–45% of cases—mirroring the mutational landscape of human breast cancer [19,20]. The *PIK3CA* H1047R hotspot mutation results in constitutive activation of the PI3K signaling pathway, whereas *BRAF* V596E (equivalent to the human V600E hotspot; canine *BRAF* numbering reflects the shorter canine protein sequence) drives oncogenic MAPK pathway signaling [21,22]. Both mutations serve as therapeutic targets and prognostic markers, and their conservation with human orthologous mutations provides significant translational value for comparative oncology research [23,24]. *KRAS* mutations, particularly the G12C hotspot, are among the most prevalent oncogenic drivers across multiple cancer types in both humans and dogs [21,22]. The *KRAS* G12C mutation (GGT → TGT) results in constitutive activation of the RAS-MAPK signaling pathway and proliferation in the driving tumor. Given the clinical significance of *KRAS* mutations and the availability of targeted therapies, sensitive detection of *KRAS* G12C in liquid biopsy specimens is of considerable interest for both human and veterinary oncology.

While CRISPR technologies have fundamentally transformed gene and cell therapy applications [25], their extraordinary sequence specificity is now being increasingly harnessed for advanced molecular diagnostics, exemplified by recent high-sensitivity platforms like MUTE-Seq that leverage engineered high-fidelity Cas9 proteins [26]. CRISPR-based wild-type depletion has been previously explored for rare mutation enrichment. Building on this concept, we hypothesized that the sequence specificity of high-fidelity CRISPR-EspCas9 could be further increased by the application of engineered guide RNAs with designed additional mismatches. Recent CRISPR-based diagnostic studies have highlighted that substrate design, including amplicon length, PAM positioning, mismatch placement, and simplified sample processing, can strongly affect single-base discrimination and translational applicability. It was recently shown that CRISPR could be designed for target recognition and signal amplification, and can also be utilized for direct lysate analysis [27]. In this study, we demonstrate that the sequential CRISPR-EspCas9 IVC workflow can be utilized as a pre-analytical enrichment step that selectively depletes wild-type DNA before downstream NGS analyses.

Unlike CRISPR applications focused on genome editing, our approach employs in vitro cleavage (IVC) using EspCas9 (enhanced-specificity SpCas9) to preferentially digest wild-type templates while leaving mutant DNA intact [28,29]. We anticipated that sequential rounds of IVC would compound enrichment effects. While a single IVC round achieves modest enrichment, iterative depletion of remaining wild-type DNA should yield exponential gains, as the relative abundance of mutant DNA increases with each round—potentially achieving enrichment factors unattainable with single-step methods.

This “negative selection” strategy is predicated on EspCas9, an engineered high-specificity SpCas9 variant developed to reduce off-target and mismatch-tolerant cleavage compared with conventional SpCas9 [30]. However, the enhanced-specificity variant Cas9 is not sufficient to provide single-base discrimination depending on mismatch position and local sequence context [31]. To overcome the specificity limitation, we sought to further enhance the target specificity to single-base precision by combining an RNA design approach with EspCas9. Our previous studies have demonstrated that incorporating an intentional double mismatch into the guide RNA significantly enhances allele-specific discrimination and detection sensitivity for low-frequency single-base mutations, such as in *EGFR* [32]. The design of allele-specific gRNAs is critical for achieving high discrimination between wild-type and mutant sequences. In this context, the key design variables for sequential IVC include amplicon length, PAM positioning, placement of the mutation within the seed region, intentional mismatch position, and compatibility with fragmented cfDNA. Computational approaches may further support gRNA design by optimizing allele specificity while minimizing off-target effects [33].

In the biosensing field, substantial efforts have been made to detect low-frequency tumor mutations in liquid biopsy samples using highly sensitive molecular readouts, including droplet digital PCR (ddPCR), UMI-based error-corrected sequencing, Safe-SeqS, and CAPP-Seq [14,34,35]. These approaches improve analytical sensitivity mainly through molecular counting, digital partitioning, or sequencing-error suppression. CRISPR-mediated wild-type depletion provides a complementary strategy because it physically reduces the excess wild-type DNA background before downstream detection. Therefore, sequential CRISPR-EspCas9 IVC can be considered a programmable pre-analytical enrichment module that may be coupled with established biosensing readouts such as NGS or digital PCR to improve rare-mutation detection in canine liquid biopsy. In this study, we demonstrated a sequential IVC method based on engineered CRISPR protein and RNA components for highly selective enrichment of mutant alleles. By targeting *PIK3CA* H1047R, *BRAF* V596E, and *KRAS* G12C mutations, we found that detection sensitivity could be improved in a target-dependent manner, reaching 0.001% MAF for *PIK3CA* and 0.01% MAF for *BRAF* under synthetic-template conditions.

## 2. Materials and Methods

### 2.1. Guide RNA Design

Guide RNAs were designed to target wild-type sequences at the *PIK3CA* H1047R (c.3140A>G; p.His1047Arg), *BRAF* V596E (c.1786T>A; p.Val596Glu; equivalent to human V600E) and *KRAS* G12C (c.34G>T; p.Gly12Cys) mutation sites. Sequences were selected to position the mutation within the seed region (positions 1–12 proximal to PAM), maximizing discrimination between wild-type and mutant templates. gRNA design was performed manually based on PAM-proximal seed-region positioning and intentional-mismatch-based allele-discrimination principles. The previously reported ARROW study was used as conceptual guidance for mismatch placement rather than as a software tool for automated sgRNA generation [33]. For each target, candidate sgRNAs were selected by considering PAM availability, mutation position relative to the PAM-proximal seed region, intentional mismatch position, and local sequence composition. Because allele discrimination by mismatch-engineered CRISPR systems is strongly affected by both mismatch position and mismatch identity, the final sgRNAs were selected empirically by in vitro cleavage screening. For the final selected sgRNAs, the 20-nt protospacer GC contents were 50% for *PIK3CA*, 45% for *BRAF*, and 60% for *KRAS*.

In designing double-mismatch sgRNAs, we considered four sequence-level features: the position of the mutation-associated mismatch within the PAM-proximal seed region, the position of the intentional mismatch, the identity of the mismatch pair, and the local sequence composition, including GC content and PAM-proximal bases. These parameters were used as design considerations rather than as a predictive model because only a limited number of mismatch-engineered sgRNA candidates were screened for each target in this study. The summary of the selected sgRNA design features is provided in Supplementary Table S1. Also, the graphical illustrations of the wild-type sequence, mutant sequence, selected sgRNA, PAM, PAM-proximal seed region, mutation-associated mismatch, and intentional mismatch for each target are provided in Supplementary Figure S1.

### 2.2. sgRNA Synthesis by In Vitro Transcription

Single guide RNAs (sgRNAs) were synthesized by in vitro transcription. DNA oligonucleotides encoding the target-specific guide sequences and scaffold oligonucleotide were purchased from Bionics Co., Ltd. (Seoul, Republic of Korea). A DNA oligonucleotide encoding the target-specific guide sequence was annealed with a scaffold oligonucleotide and extended to generate a double-stranded DNA template containing the T7 promoter, guide sequence, and sgRNA scaffold. For the extension reaction, 1 μL DNA oligonucleotide, 1 μL scaffold oligonucleotide, 10 μL 2× KOD Master Mix (TOYOBO bio, Osaka, Japan), and 8 μL nuclease-free water were mixed in a total volume of 20 μL. The extension reaction was performed under the following cycling conditions: 98 °C for 30 s; 25 cycles of 98 °C for 10 s, 55 °C for 5 s, and 68 °C for 2 s; followed by 68 °C for 5 s and hold at 4 °C. The extended products were purified using a DNA clean-up kit (DNA clean and concentrator, Zymo Research, Irvine, CA, USA) and the DNA amount was measured by fluorometry using a Qubit fluorometer (Thermo Fisher Scientific, Waltham, MA, USA). Up to 1000 ng of purified double-stranded DNA template was used for in vitro transcription; this value refers to the total amount of DNA template used for transcription, not the final template concentration. Transcription was carried out at 37 °C overnight using T7 RNA polymerase (New England Biolabs, Ipswich, MA, USA) according to the manufacturer’s instructions. Following transcription, sgRNAs were purified using a commercial RNA purification kit (RNA clean and concentrator, Zymo Research). Briefly, RNA-binding buffer and ethanol were added to the transcription reaction, and the sample was loaded onto a silica membrane column. The column was washed with RNA prep buffer and RNA wash buffer, and purified sgRNA was eluted in DNase/RNase-free water. The purified sgRNAs were quantified by fluorometry using Qubit and stored at −80 °C until use.

### 2.3. Expression and Purification of EspCas9 Protein

The EspCas9 expression plasmid (EspCas9 e1.0) was transformed into *Escherichia coli* BL21 (DE3) by heat shock, including ice incubation, heat shock at 42 °C for 1 min, and recovery in SOC medium (Super Optimal broth with Catabolite repression) at 37 °C for 30–60 min. Transformants were selected on LB (Lysogeny Broth) agar plates containing 50 μg/mL of kanamycin overnight at 37 °C. A single colony was used for preculture in 10 mL LB medium supplemented with 50 μg/mL kanamycin overnight at 37 °C, followed by inoculation into two 250 mL main cultures (4 mL preculture per flask). When the optical density at 600 nm (OD600) reached 0.5–0.6, protein expression was induced with 0.5 mM isopropyl β-D-1-thiogalactopyranoside (IPTG), and cultures were incubated at 18 °C overnight. Cells were harvested by centrifugation at 4000–5000 rpm for 10 min at 4 °C. Pellets were resuspended in lysis buffer supplemented with 1 mM phenylmethylsulfonyl fluoride (PMSF) and 1 mM dithiothreitol (DTT) and lysed by sonication (3 s on/9 s off, 30% amplitude, on ice). Lysates were clarified by centrifugation at 11,500 rpm for 30 min (4 °C) and filtered through a 0.45 μm filter. EspCas9 was purified using Ni–NTA affinity chromatography (4 mL resin), including equilibration, wash, and elution steps according to the laboratory standard protocol (Supplementary Table S2). Eluted fractions were concentrated and exchanged into storage buffer, and protein concentration was measured by A280 using a NanoDrop spectrophotometer (Thermo Fisher Scientific, Waltham, MA, USA) and stored at −80 °C. Expression and purification quality were monitored by sodium dodecyl sulfate–polyacrylamide gel electrophoresis (SDS–PAGE) (Supplementary Figure S2).

### 2.4. In Vitro DNA Cleavage (IVC)

For each IVC reaction, EspCas9–sgRNA ribonucleoprotein complexes were assembled by incubating 1.22 µg EspCas9 protein with target-specific sgRNA in cleavage buffer (20 mM Tris-HCl, 100 mM NaCl, 10 mM MgCl2, 1 mM DTT) for 10 min at 25 °C. The sgRNA amount was 100 ng for *PIK3CA* and *KRAS*, and 300 ng for *BRAF*. After RNP assembly, 30 ng DNA template was added, and the final reaction volume was adjusted to 30 µL. Reactions were incubated at 37 °C for 60 min and terminated by addition of 1 µL Proteinase K (20 mg/mL; Enzynomics, Daejeon, Republic of Korea), followed by incubation at room temperature for 10 min. These inputs correspond to approximately 254 nM EspCas9 and approximately 105 nM sgRNA for *PIK3CA*/*KRAS* or 315 nM sgRNA for *BRAF*. Detailed mass and molar ratios are provided in Supplementary Table S3.

### 2.5. Sequential IVC Protocol

For sequential enrichment, the following protocol was employed:

1. Round 1: Initial PCR amplification (30 cycles, to normalize template to 30 ng) → column purification → 1st IVC.
2. Round 2: Semi-nested PCR amplification of Round 1 product (30 cycles) → column purification → 2nd IVC.
3. Round 3: Semi-nested PCR amplification of Round 2 product (30 cycles) → column purification → 3rd IVC → NGS library preparation (adaptor PCR + index PCR), which also serves as the PCR amplification step for the third round of enrichment.

To maintain amplification specificity across sequential rounds, a semi-nested PCR strategy was employed (Supplementary Figure S3): forward primers were shortened by approximately 5–10 bp for the round 2 and round 3 PCR amplifications (primer sequences provided in Supplementary Table S4). Between rounds, DNA concentration was normalized to ensure consistent input for each IVC step. PCR amplification was performed using 2× KOD Master Mix (TOYOBO Bio) with the primers listed in Supplementary Table S4. Each PCR reaction contained 10 μL of 2× KOD Master Mix, 1 μL of forward primer working solution (10 μM), 1 μL of reverse primer working solution (10 μM), and 30 ng DNA template in a total volume of 20 µL. The PCR cycling conditions were as follows: initial denaturation at 98 °C for 30 s, 30 cycles of denaturation at 98 °C for 10 s, annealing at 55 °C for 5 s, and extension at 68 °C for 1 s, followed by final extension at 68 °C for 5 s. PCR amplification was used to generate sufficient DNA template before each IVC round and to amplify the remaining un-cleaved DNA after IVC. After each PCR step, DNA products were purified using a DNA clean and concentrator kit (Zymo Research) according to the manufacturer’s protocol. Briefly, DNA was bound to a silica membrane column, washed to remove residual primers, enzymes, and salts, and eluted in 20 µL of nuclease-free water before the next IVC reaction.

### 2.6. Preparation of Genomic and Cell-Free DNA from Canine Cancer Cell Lines

Genomic DNA was extracted from a *PIK3CA* H1047R-positive canine mammary gland tumor cell line (MT, MGT 1) and from a nominally wild-type canine cell line (WT, MGT 8). Cells were cultured under standard conditions, and gDNA was extracted using a commercial kit (QIAamp DNA Mini Kit; Qiagen, Hilden, Germany). MT and WT gDNA were mixed, and serial 10-fold dilutions were prepared. Each mixture was subjected to a single round of IVC. Enrichment was assessed by Sanger sequencing and by next-generation sequencing (NGS) for precise MAF quantification.

To evaluate IVC performance in a more biologically relevant context, a cfDNA mimic was prepared from conditioned culture medium. *PIK3CA* H1047R canine cancer cells (MT, MGT 1) and wild-type canine cells (WT, MGT 8) were cultured in serum-free medium for 72 h. Supernatant was collected, centrifuged to remove cellular debris (2000× *g*, 10 min), and cell-free DNA was extracted using a cfDNA-optimized extraction kit (MagListo cfDNA Extraction Kit; Bioneer, Daejeon, Republic of Korea). The MT cfDNA mimic was diluted serially in WT cfDNA mimic by sequential 10-fold dilutions, and each dilution was subjected to a single round of IVC followed by PCR amplification, Sanger sequencing, and NGS.

### 2.7. Synthetic DNA Template Preparation

Wild-type and mutant DNA templates for *PIK3CA* H1047R, *BRAF* V596E, and *KRAS* G12C targets were generated by PCR amplification from 500 bp synthetic gene fragments (Integrated DNA Technologies, Coralville, IA, USA). Each fragment was designed with a common flanking sequence on both ends to enable the use of a shared primer set across all three targets during the semi-nested PCR rounds (Rounds 1–3). The central region of each fragment contained the target-specific sequence for *PIK3CA*, *BRAF*, or *KRAS*, with separate fragments synthesized for the wild-type and mutant alleles of each target. This shared-flank design allowed target-specific differentiation only at the adaptor PCR step, where a target-specific reverse primer (*PIK3CA*_deep_R, *BRAF*_deep_R, *KRAS*_deep_R) was used in combination with a common forward primer (deep_F). Wild-type and mutant DNA templates were mixed gravimetrically at defined ratios (100:0, 90:10, 99:1, 99.9:0.1, 99.99:0.01, 99.999:0.001, and 0:100), ensuring accurate control of input mutant allele fractions. These 500 bp synthetic templates were used to provide controlled spike-in materials for analytical evaluation. They were not intended to reproduce the fragment-size distribution of plasma cfDNA.

### 2.8. Sanger Sequencing

PCR amplicons were purified and submitted for bidirectional Sanger sequencing (Bionics Co., Ltd., Seoul, Republic of Korea). Returned chromatograms were aligned to the canine *PIK3CA* reference sequence using SnapGene software (v 8.2.2) to identify mutations at the H1047R site. Base calling was performed by the automated algorithm of the sequencing platform. At positions where minor secondary peaks were observed below the automated calling threshold, chromatograms were visually inspected to assess the presence of low-level mutant signals.

### 2.9. Short-Amplicon Validation for cfDNA-Compatible Assay Design

To evaluate whether the IVC strategy could be adapted to a shorter amplicon format, an additional forward primer (PIK3CA_deep_short_F) was designed and used together with the original reverse primer (PIK3CA_deep_R) to amplify a shorter *PIK3CA* target region of 170 bp. The shorter amplicon retained the same mutation site and the PAM-proximal guide RNA target region used for the original assay. The 170 bp amplicon was generated from the cfDNA mimic prepared from the *PIK3CA* H1047R-positive MGT 1 cell line, subjected to one round of IVC enrichment, and analyzed by Sanger sequencing. Relative peak signals at the mutation site were quantified from the Sanger chromatograms. The primer sequences are listed in Supplementary Table S4.

### 2.10. Next-Generation Sequencing

Following the final (3rd) IVC, NGS libraries were prepared directly from IVC products by adaptor PCR to append sequencing adapters, followed by index PCR to add sample-specific dual indices. As the adaptor PCR directly amplifies DNA surviving the third IVC round, this step effectively serves as the PCR amplification component of the third enrichment round. For the main analytical spike-in datasets, sequencing was performed on an Illumina NovaSeq X Plus platform (Illumina, Inc., San Diego, CA, USA) using a 2 × 150 bp paired-end protocol, targeting > 100,000 reads per sample; the actual sequencing depth achieved for these datasets was approximately 6–10 million reads per sample. The additional independent *PIK3CA* validation dataset was sequenced separately on an Illumina iSeq 100 (Illumina, Inc., San Diego, CA, USA) system. Because this validation run had lower read depth than the main NovaSeq dataset, it was used to confirm the round-dependent enrichment trend rather than to redefine the analytical LOD. Raw sequencing reads were adapter-trimmed and quality-filtered using Trimmomatic (v0.39) with a sliding window approach (4-base average Phred quality score Q > 15). The processed high-quality reads were subsequently aligned to the reference sequences using HISAT2. Post-alignment processing, including SAM-to-BAM conversion, sorting, and indexing, was conducted using Samtools. Mutant allele frequency was calculated as mutant reads/total mapped depth × 100%, where total mapped depth comprises wild-type, mutant, and other-base reads at the target position.

### 2.11. Statistical Analysis

Spike-in enrichment experiments were initially performed as single measurements per condition. For the key *PIK3CA* sequential IVC experiment, an independent repeat experiment was additionally performed through the full IVC–PCR–NGS workflow, and the replicate dataset is provided in Supplementary Figure S4. Because the additional *PIK3CA* validation dataset was generated at lower read depth, it was used to support the reproducibility of the round-dependent enrichment trend rather than to redefine the analytical LOD. For the main high-depth NGS datasets, deep sequencing at approximately 6–10 million reads provided substantial statistical power for read-level comparisons, although it does not replace independent biological replication.

Enrichment fold was calculated as the mutant allele frequency after each IVC round divided by the measured mutant allele frequency in the matched negative control (NC, gRNA without EspCas9) at the corresponding input concentration. Odds ratios (ORs) and exact *p*-values for read-count-level comparisons were computed using SciPy v1.11 in Python 3.11 with scipy.stats.fisher_exact. Data visualization was performed using GraphPad Prism v11.0. For *PIK3CA* validation analysis, the MAF values from NGS of the two biological replicates were shown with mean ± SD in Supplementary Figure S4. Statistical comparisons between the sgRNA-only negative control and third-round IVC values were performed using unpaired *t*-tests after log10 transformation in GraphPad Prism. Read-count-level Fisher’s exact test was used for analytical LOD determination based on the NGS dataset, as summarized in Table 1.

## 3. Results

### 3.1. Schematics of Sequential CRISPR Enrichment Strategy

**Figure 1:**
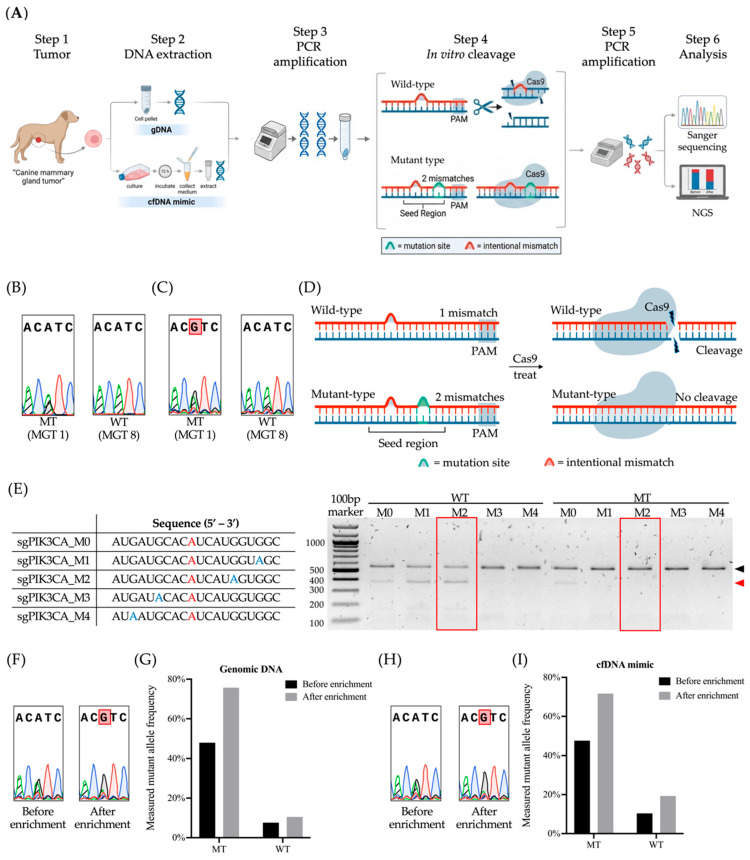
Validation of allele-specific CRISPR-EspCas9 enrichment using genomic DNA (gDNA) and cell-free DNA (cfDNA) mimic for *PIK3CA* H1047R. (**A**) Workflow for *PIK3CA* H1047R enrichment using genomic DNA (gDNA) and cell-free DNA (cfDNA) mimic from canine mammary gland tumor cell lines. The workflow indicates DNA extraction, initial PCR amplification, IVC-mediated wild-type depletion, post-IVC PCR amplification of the remaining un-cleaved DNA, and downstream Sanger sequencing or NGS analysis; (**B**,**C**) PCR-amplified *PIK3CA* templates from mutant (MGT 1) and wild-type (MGT 8) cell lines using (**B**) gDNA and (**C**) cfDNA mimic, used for subsequent IVC; (**D**) Design of allele-specific sgRNA featuring an intentional mismatch to maximize discrimination between wild-type and mutant sequences; (**E**) In vitro cleavage (IVC) screening of candidate sgRNA variants (M0–M4) for the *PIK3CA* target. Red letters indicate the mutation site, and blue letters indicate the intentional mismatch. Black arrows denote un-cleaved DNA, while red arrows highlight cleaved DNA fragments. The red box indicates the selected sgPIK3CA_M2 variant used for subsequent experiments; (**F**) Analyses of the mutation ratios of the *PIK3CA* H1047R mutation in the genomic DNAs before and after IVC enrichment by Sanger chromatogram. (**G**) Quantification of *PIK3CA* H1047R mutant ratios of the genomic DNAs by NGS analyses. (**H**) Analyses of the mutation ratios of the *PIK3CA* H1047R mutation in the cell-free DNA mimics before and after IVC enrichment by Sanger chromatogram. (**I**) Quantification of *PIK3CA* H1047R mutant ratios of the cell-free DNA mimics by NGS analyses.

We sought to address the challenges of detection limits that are inherent to liquid biopsy. To this end, we developed a sequential CRISPR-EspCas9-based workflow involving multiple rounds of wild-type DNA depletion (Figure 1A). The underlying principle builds upon the established approach of selective wild-type cleavage but introduces iterative processing to compound enrichment effects. We designed modified guide RNAs targeting wild-type sequences at the *PIK3CA* H1047R (CAT → CGT), *BRAF* V596E (GTG → GAG), and *KRAS* G12C (GGT → TGT) mutation sites. The guide RNAs were positioned such that tumor-associated mutations fall within the seed region—the PAM-proximal sequence critical for EspCas9 cleavage specificity. This configuration ensured efficient cleavage of wild-type templates while single-nucleotide mismatches with mutant sequences substantially impaired EspCas9 activity. The sequential workflow comprised the following steps: (1) DNA template preparation with defined wild-type: mutant ratios; (2) initial PCR amplification to normalize template amount; (3) the first round of IVC; (4) semi-nested PCR amplification; (5) the second round of IVC; (6) semi-nested PCR amplification; (7) the third round of IVC; (8) NGS library preparation (adaptor and index PCR, which also serves as the amplification step for the third round of enrichment); (9) NGS quantification.

### 3.2. Application of the CRISPR Enrichment for Enhanced Detection of a PIK3CA Mutation in Genomic DNA and cfDNA Mimic from Canine Cancer Cells

To evaluate IVC performance in a more biologically relevant context, we prepared the genomic DNA (gDNA) and cfDNA mimic from canine mammary gland tumor cell lines. To this end, the gDNA was extracted directly from cell pellets of a *PIK3CA* H1047R-positive cell line (MGT 1, mutant) and a wild-type cell line (MGT 8). A cell-free DNA (cfDNA) mimic was prepared from the conditioned culture medium of the same cell lines (Figure 1A). Sanger chromatograms of PCR-amplified templates from gDNA (Figure 1B) and cfDNA mimic (Figure 1C) confirmed the presence of the expected wild-type and mutant sequence patterns at the *PIK3CA* H1047R site.

In Figure 1B, PCR-amplified gDNA from both MGT 1 and MGT 8 produced clean Sanger chromatograms around the *PIK3CA* H1047R site. The mutation-positive MGT 1 sample showed a mutation-associated G signal at the expected position, whereas the MGT 8 sample showed a dominant wild-type A peak with no obvious mutant peak. In Figure 1C, PCR-amplified cfDNA mimic samples also showed interpretable chromatograms. However, in the MGT 8 cfDNA mimic sample, a small G peak was observed together with the dominant wild-type A peak at the mutation site. Although this minor peak could not be quantified reliably by Sanger sequencing alone, it was consistent with the low-level *PIK3CA* H1047R signal detected by NGS in the MGT 8 cfDNA mimic sample.

To aid interpretation of the minor mutation-associated peak observed in the MGT 8 samples, an additional representative Sanger chromatogram from a PCR-amplified *PIK3CA* H1047R-positive synthetic DNA template is provided in Supplementary Figure S5A. This chromatogram shows the expected position and peak pattern of the mutation-associated G signal at the *PIK3CA* H1047R site and provides a reference for interpreting the minor G peak observed in the MGT 8 cfDNA mimic sample. Together with the low-level *PIK3CA* H1047R signal detected by NGS, this supports the interpretation that the minor G peak is consistent with a low-level mutant-like signal rather than solely random Sanger background.

To achieve allele-specific cleavage, we designed engineered single-guide RNAs (sgRNAs) with intentional mismatches within the sgRNA sequence targeting the *PIK3CA* H1047R mutation site. The designed sgRNAs contained one mismatch against the wild-type sequence, permitting EspCas9 cleavage. On the other hand, the *PIK3CA* H1047R mutant sequence harbored additional mismatches at the mutation site, and the two combined mismatches selectively inhibited the cleavage of the mutant allele (Figure 1D). Among the tested sgRNA variants (M0–M4), we found that the M2 sgRNA efficiently cleaved wild-type DNA while the mutant DNA remained virtually un-cleaved and utilized M2 for the subsequent experiments for the allele-specific discrimination of the *PIK3CA* H1047R mutation (Figure 1E).

Application of IVC enrichment to MT (MGT 1) and WT (MGT 8) gDNA and cfDNA mimic samples resulted in a marked increase in the mutant G peak relative to the wild-type A peak for both gDNA (Figure 1F) and cfDNA mimic (Figure 1H), indicating effective enrichment of the mutant allele. NGS quantification corroborated these findings, where, for the MT sample, the MAF increased from 48.0% to 75.7% (gDNA, Figure 1G) and from 47.7% to 71.7% (cfDNA mimic, Figure 1I) relative to the negative control. Notably, the quantification by NGS also revealed a baseline MAF of 7.1% (gDNA) and 9.9% (cfDNA mimic) in the nominally wild-type MGT 8 cell line, suggesting that Sanger sequencing had called the MGT 8 cell line wild-type despite the low mutation signal. The detectable increase in MAF in MGT 8 cells after IVC enrichment suggested that a low-level *PIK3CA* mutant-like signal was present in the cell line. The results demonstrated that the combination of IVC enrichment with Sanger sequencing and NGS can improve the detection of low-level mutations.

Clinical plasma cfDNA is highly fragmented, with a major mononucleosomal peak at approximately 167 bp in humans and a similar peak around 163 bp reported in dogs [36]. We therefore sought to assess the efficacy of the IVC enrichment on a shorter cfDNA-sized template. To this end, we generated a 170 bp *PIK3CA* H1047R amplicon from the cfDNA mimic prepared from the *PIK3CA* H1047R-positive MGT 1 cell line and subjected it to one round of IVC enrichment. Sanger chromatogram analysis showed an increase in the mutant-associated G signal after enrichment, with a corresponding decrease in the wild-type-associated A signal (Supplementary Figure S5B). The results suggested that CRISPR IVC-mediated allele-selective enrichment can be applied to mononucleosomal cfDNA.

We next asked if the IVC method by double-mismatch sgRNAs could enhance the detection limit of low MAF. To this end, we tested the method in serial 10-fold dilutions of the mutant DNA in wild-type DNA. While MAF consistently increased after enrichment compared to negative controls across the dilution series, the elevated baseline in the wild-type reference (7.1–9.9%) limited reliable quantification at low input fractions. Therefore, reliable quantification at input fractions below 0.5% was limited in this cell-line-based dilution setting, and synthetic templates with defined mutant allele fractions were used for subsequent analytical sensitivity testing (Supplementary Figure S6).

### 3.3. Quantitative Analyses of EspCas9-Based IVC Enrichment on Cancer-Associated Single-Base Mutant DNAs at Low MAFs

We next sought to quantitatively analyze the enrichment performance of IVC at sub-percent input levels, without interference from endogenous mutations. To this end, we assessed the enrichment MAF by IVC on synthetic DNA templates with defined ratios of mutant-to-wild DNA. We prepared synthetic 500 bp DNA fragments encoding wild-type and mutant *PIK3CA* alleles at defined ratios of 100%, 10%, 1%, 0.1%, 0.01%, 0.001%, and 0%.

**Figure 2:**
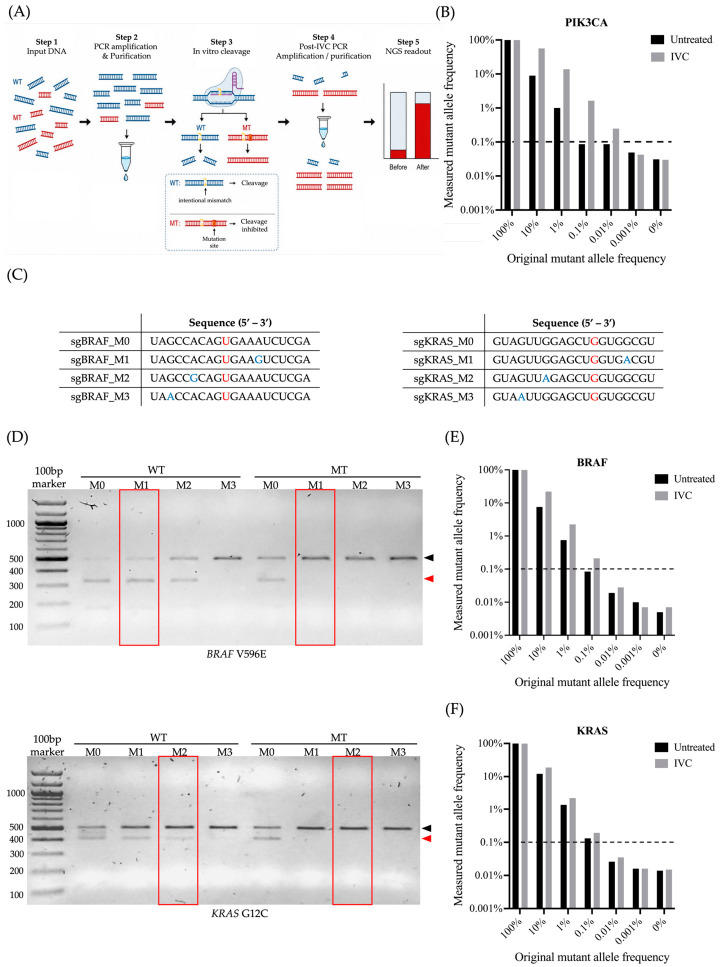
Single-round IVC enrichment with synthetic *PIK3CA*, *BRAF*, and *KRAS* templates. (**A**) Schematic of the single-round IVC workflow. Synthetic DNA templates containing wild-type and mutant sequences were mixed at defined MAF ratios, amplified by PCR, subjected to IVC-mediated wild-type depletion, re-amplified by PCR, and analyzed by NGS. Blue and red DNA fragments indicate wild-type and mutant alleles, respectively. Yellow and orange marks indicate the intentional mismatch and mutation-site mismatch, respectively; (**B**) NGS quantification of detected MAFs for IVC-treated and untreated samples of *PIK3CA* H1047R MAF across the various original MAF ratios; (**C**) Sequences of sgRNA candidate mismatch sgRNAs for *BRAF* V596E and *KRAS* G12C mutations. M0 presents a perfect-match sgRNA to the wild-type sequence, while M1–M3 contain a single intentional mismatch. The red and blue letters indicate the original and the additional mismatch positions, respectively; (**D**) In vitro cleavage of *BRAF* V596E and *KRAS* G12C mutant DNAs with the designed sgRNAs. Black and red arrows indicate un-cleaved and cleaved DNA fragments, respectively. The red box indicates the selected sgRNA variant used for subsequent experiments; (**E**,**F**) NGS quantification of mutant allele frequency after IVC enrichments for (**E**) *BRAF* and (**F**) *KRAS*. The dashed horizontal line indicates the approximate NGS background threshold of 0.1% MAF.

Using sgPIK3CA-M2, which showed selective cleavage of *PIK3CA* H1047R, we conducted IVC enrichment on the synthetic *PIK3CA* samples with a series of MAFs (Figure 2A). We then compared the detected MAFs before and after IVC enrichment via NGS quantification (Figure 2B). We found that the detected MAFs were significantly increased after IVC, where the originally prepared MAFs were 0.01% and above (Figure 2B). Notably, in the sample prepared with 0.01% MAF, we observed that IVC enrichment resulted in an increase in detected MAF well above 0.1%, which is generally considered the detection limit of NGS due to background signals.

We next asked if the IVC enrichment could also be applied to other cancer-related point mutations: *BRAF* V596E and *KRAS* G12C. For each target, we designed sgRNAs with mismatches by in vitro cleavage (Figure 2C). Three mismatch variants (M1–M3) were tested for each target alongside the wild-type-specific perfect-match sgRNA (M0). In vitro cleavage analyses followed by gel electrophoresis showed that sgBRAF-M1 and sgKRAS-M2 efficiently cleaved wild-type DNA, while cleavages were undetectable in mutant DNAs (Figure 2D). The selected sgRNAs were then applied to a series of DNA samples with various ratios of corresponding *BRAF* and *KRAS* mutant to wild-type alleles, ranging from 0% to 100%. NGS quantification showed significant enrichment of detected MAF for both *BRAF* V596E and *KRAS* G12C samples with original MAFs at and above 0.1% (Figure 2E,F). These results suggested that the IVC enrichment method could be generally applied for improved MAF detection of various single-base mutations.

### 3.4. Improving the MAF Detection Sensitivities by Iterative IVC Enrichments

**Figure 3:**
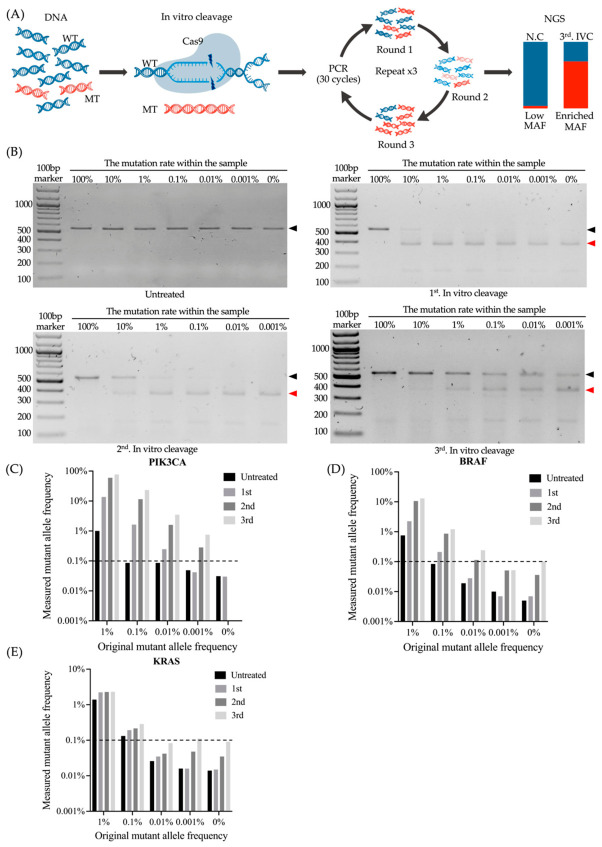
Sequential CRISPR-EspCas9 IVC showed a progressive increment of detected MAFs. (**A**) Schematic of the iterative workflow involving three successive rounds of IVC and intervening semi-nested PCR amplifications; (**B**) Agarose gel electrophoresis of *PIK3CA* IVC products over successive rounds for the *PIK3CA* target. Black and red arrows indicate un-cleaved and cleaved DNA fragments, respectively; (**C**–**E**) NGS quantification of MAFs after each round of IVC for (**C**) *PIK3CA* H1047R, (**D**) *BRAF* V596E, and (**E**) *KRAS* G12C samples with original MAFs from 0% to 1%.

We next sought to further increase the efficacy of the single-round IVC enrichment and asked how sequential IVC may enhance the enrichment efficiencies (Figure 3A). For experimental assessment of the scheme, we again prepared the synthetic DNA samples with various original mutant allele frequencies (MAF) and quantified the detected MAFs after one, two, or three iterations of IVC enrichments, where CRISPR-based wild-type depletion was followed by PCR amplification between each round.

Agarose gel electrophoresis revealed progressive depletion of wild-type DNA with each successive IVC round (Figure 3B, Supplementary Figure S7). For the *PIK3CA* 0% background sample, no visible PCR product was obtained after the second PCR step following IVC, leaving no recoverable intact DNA band for purification or library preparation. The corresponding gel image is provided in Supplementary Figure S8. We observed that un-cleaved DNA bands were more prominent in the second and third IVC iterations, compared to the first IVC round, suggestive of sequential increment of mutant DNA. Notably, the 100% mutant DNA sample remained intact across all three rounds, suggesting that sequential processing did not significantly degrade the mutant templates. NGS quantification showed the compounding enrichment with sequential IVC across successive rounds for *PIK3CA*, *BRAF*, and *KRAS* mutations (Figure 3C–E, Supplementary Figure S9). *PIK3CA* H1047R demonstrated the prominent progressive depletion of wild-type DNA (Table 2). For 0.1% input, the detected MAF increased to 23.48% after the sequential enrichment, indicating approximately 160-fold cumulative enrichment. We also observed ~15-fold enrichment of MAF detection in the ultra-low 0.001% input level, resulting in ~0.75% MAF, which is well above the general NGS baseline noise level of ~0.1%. As a negative control, we also analyzed samples that were incubated with sgRNA only, without EspCas9. As anticipated, quantification of the sgRNA-only (named untreated) samples showed no meaningful enrichment of MAFs above the ~0.1% background NGS noise level. To assess the reproducibility of the central *PIK3CA* result, we performed an independent repeat experiment using the same sequential IVC-PCR-NGS workflow. The original and repeated datasets showed a similar round-dependent increase in measured MAF (Supplementary Figure S4). In the combined mean ± SD summary of the original and additional *PIK3CA* NGS datasets, third-round IVC increased the measured MAF compared with the sgRNA-only negative control at mutant-containing input concentrations, with statistical significance indicated in Supplementary Figure S4B.

We also assessed the background MAF values from 0% mutant controls during sequential IVC (Supplementary Table S5). For all three target sequences, the MAF of 0% background remained below 0.1%, which is considered the NGS noise level. Moreover, later-round *PIK3CA* 0% controls did not produce a detectable PCR product following IVC, suggesting that the wild-type DNA was completely cleaved. (Supplementary Figure S8). In contrast, *BRAF* and *KRAS* retained residual PCR products, allowing round-matched 0% background MAF values to be estimated.

*BRAF* V596E showed consistent progressive enrichment across all three rounds (Table 3), achieving a cumulative 15-fold increase (reaching 1.22% MAF) at a 0.1% initial input concentration.

*KRAS* G12C exhibited ~1.7-fold enrichment at 1% and ~2.2-fold enrichment at 0.1% input (Table 4).

We further asked if sequential IVC generated a false-positive enrichment of signals in 0% mutant controls. To this end, we analyzed the 0% background MAF values for each target and IVC round (Supplementary Table S5). For *PIK3CA*, the 0% MAF value from the sgRNA-only negative control was initially 0.031% and was maintained at 0.030% after the first IVC round. After the second IVC round, the 0% MAF of *PIK3CA* was completely cleaved, suggesting that virtually all alleles were wild type (Supplementary Figure S8). For *BRAF* and *KRAS*, MAF values could be calculated across sequential IVC rounds, allowing round-matched 0% background MAF values to be estimated. These values remained at or below the estimated NGS background level of approximately 0.1%: 0.102% for *BRAF* and 0.091% for *KRAS* after the third IVC round, respectively. The results suggested that the mutant-like signals of 0% samples mainly reflect NGS noise-level background rather than the generation of false-positive MAF signals.

## 4. Discussion

**Figure 4:**
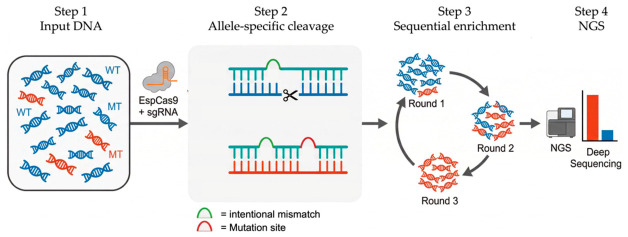
Conceptual model of enrichment through sequential IVC. Schematic illustration of the enrichment principle. Each round of IVC selectively removes a fraction of remaining wild-type DNA, producing multiplicative gains in mutant allele frequency across successive rounds. Blue and red DNA fragments indicate wild-type and mutant alleles, respectively. Green and red marks indicate the intentional mismatch and mutation site, respectively. Arrows indicate the sequential IVC workflow.

Our results demonstrate that sequential CRISPR-EspCas9 IVC achieves enrichment factors far exceeding those of single-step methods. This study does not introduce CRISPR-based enrichment as a new concept but rather demonstrates a sequential EspCas9-based implementation with target-dependent performance. Iterative wild-type depletion (Figure 4) produces compounding gains in mutant representation. Notably, enrichment efficiency varied substantially across targets, indicating that guide RNA design and sequence context are critical determinants of performance and should be considered when applying the approach to additional mutations.

Consistent with this model, sequential IVC progressively increased the mutant allele fraction for *PIK3CA* H1047R from a low-input sample to a level readily detectable by standard sequencing. The magnitude of this effect exceeded that typically reported for single-round IVC, indicating that our optimized conditions enabled highly efficient cumulative wild-type depletion for this target.

However, repeated PCR amplification between IVC rounds may introduce bias, and low-level sequencing background can become more relevant when enrichment efficiency is limited. Although the semi-nested PCR design likely helped preserve specificity, the reduced fold-enrichment observed at lower concentrations may reflect not only IVC-mediated wild-type depletion but also stochastic amplification effects. Analyses of 0% control samples of all three targets after IVC rounds showed that the MAF values of 0% samples did not exceed the noise level of NGS (Supplementary Table S5). *BRAF* and *KRAS* 0% controls showed MAFs at or below the 0.1% noise level across sequential rounds, whereas later-round *PIK3CA* 0% controls yielded no recoverable PCR product for subsequent NGS analyses. The results suggested that MAF signals of 0% samples are noise, and it is inadequate to consider them as quantitative measurements. Therefore, we conclude that the MAF values of *KRAS* G12C are actually enriched, albeit modestly, by IVC rounds since the seemingly decreasing *KRAS* odds ratio might be compounded by noise fluctuation of the 0% background sample. Future clinical implementations should therefore incorporate UMI-based error correction or orthogonal validation methods to better distinguish true enrichment from amplification- or sequencing-derived noise.

This study demonstrated an analytical sensitivity of 0.001% under synthetic template conditions for *PIK3CA* and 0.01% for *BRAF*. The sub-single-copy concern previously raised for 0.001% detection does not apply to synthetic spike-in templates: unlike genomic DNA, where 30 ng corresponds to ~9000 genome equivalents (yielding <1 mutant copy at 0.001%), the 500 bp synthetic fragments used here provide approximately 5.5 × 10^10^ copies per 30 ng, corresponding to ~550,000 mutant copies even at 0.001% input. The LOD at this concentration is therefore not constrained by stochastic sampling of rare templates. While current liquid biopsy approaches are often more effective when tumor burden is substantial, our sequential CRISPR enrichment method may contribute to the development of early-stage detection strategies for screening asymptomatic, high-risk dogs. Clinical sensitivity with actual cfDNA may differ owing to DNA fragmentation and sample complexity. Furthermore, the 0% control and negative control served distinct purposes in this study: the former estimated background mutant-like signal after IVC, whereas the latter confirmed EspCas9-dependent enrichment.

### 4.1. Statistical Validation of Detection Sensitivity by Fisher’s Exact Test

To rigorously define the LOD, we applied Fisher’s exact test to compare mutant read counts at each spike-in concentration against the corresponding 0% background control or reference (Table 1). For *BRAF* and *KRAS*, round-matched 0% controls were used for each IVC round. For *PIK3CA*, later-round 0% controls were not available because no detectable PCR product was recovered for subsequent second- and third-round processing after IVC; therefore, the available first-round 0% control was used as the background reference for later-round comparisons. In this study, the analytical limit of detection (LOD) under the present experimental conditions was defined as the lowest input level that satisfied both criteria: (i) a statistically higher mutant signal than the matched 0% control, and (ii) biological interpretability under the template input conditions. Statistical significance alone was not considered sufficient when the expected mutant copy number was below one per reaction. The 0% control was used to estimate background mutant-like signal arising after the same IVC workflow, whereas the negative control (NC, gRNA without EspCas9) was used separately to confirm EspCas9-dependent enrichment at the matched input level. These two comparisons address different questions and were not used interchangeably. Given the high sequencing depth (~6–10 million reads per sample), virtually all comparisons achieved extreme statistical significance (BH-adjusted *p* < 10^−27^), making *p*-values alone uninformative for assessing practical detectability. We therefore focused on the odds ratio (OR) as the primary metric for evaluating enrichment magnitude. For *PIK3CA* H1047R, detectable enrichment was observed at 0.001% input, with OR values of 1.4 (1st), 9.5 (2nd), and 25.4 (3rd round). For the second- and third-round *PIK3CA* comparisons, these OR values were calculated using the available first-round 0% background reference because later-round 0% controls were not available. Because synthetic DNA templates were used (500 bp fragments, ~5.5 × 10^10^ copies per 30 ng input), even at 0.001% input, approximately 550,000 mutant copies were present—well above the stochastic sampling threshold. The analytical LOD for *PIK3CA* was therefore defined as 0.001% under the present synthetic template conditions, supported by an OR of 25.4 at the third round. We found that the MAF of 0% background control samples remained below 0.1%, a generally considered NGS noise level, after IVC enrichment rounds. After the second round of enrichment, the *PIK3CA* 0% controls did not produce a detectable product, suggesting that all the wild-type template DNA was cleaved and therefore could not be analyzed by NGS (Supplementary Figure S8). Therefore, the first-round 0% sample was used as the available background reference for the *PIK3CA* LOD analysis.

For *BRAF* V596E at 0.01% input, OR values versus the round-matched 0% control were 3.9 (1st), 3.2 (2nd), and 2.4 (3rd round). These results indicate analytically detectable enrichment above background, although the OR magnitude remained modest compared with *PIK3CA*. Comparison against the negative control (NC, gRNA without EspCas9) yielded OR = 47.5 at the third round, separately confirming that the observed enrichment was EspCas9-dependent. Accordingly, *BRAF* 0.01% was interpreted as meeting the analytical detection criterion, while the negative control comparison was used as supporting evidence for the mechanism rather than as an alternative LOD definition.

For *KRAS* G12C at 0.1% input, OR values against round-matched 0% controls were 13.2 (1st), 6.3 (2nd), and 3.2 (3rd round), showing a decreasing trend across sequential IVC rounds. However, the 0% background MAF values remained at or near the expected NGS background level of approximately 0.1%, suggesting that this trend is unlikely to reflect strong practical false-positive generation by sequential IVC. Instead, the decreasing OR is more likely explained by limited separation between mutant enrichment and low-level background under the current *KRAS* guide design. Comparison against the NC 0.1% control (gRNA without EspCas9 at matched input concentration) yielded OR = 2.2 at the third round, indicating EspCas9-dependent enrichment that remained modest in practical magnitude. Thus, although *KRAS* showed statistically detectable enrichment, it was not considered to achieve a robust practical LOD under the current guide design. Further optimization of sgRNA design may improve enrichment efficiency for *KRAS* targets.

These analyses define the analytical LOD as follows: *PIK3CA* H1047R, 0.001% MAF (supported by OR = 25.4 at the third round; sub-single-copy concerns do not apply to the synthetic templates used, which provide ~550,000 mutant copies at this concentration); *BRAF* V596E, 0.01% MAF (meeting the analytical detection criterion versus the matched 0% control, with NC comparison providing separate support for EspCas9 dependence). *KRAS* G12C showed statistically detectable but practically limited enrichment and was therefore not considered to achieve a robust practical LOD under the current guide design.

### 4.2. Differential Enrichment Efficiency and sgRNA Design Considerations

A clear difference in enrichment efficiency was observed between *PIK3CA* (~160×) and *BRAF* V596E (~15×). This difference likely reflects variation in guide RNA–target interactions and local sequence context. The selected 20-nt protospacers had GC contents of 50% for *PIK3CA*, 45% for *BRAF*, and 60% for *KRAS*. We anticipate that the relatively high GC-ratio of the *KRAS* target sequence may have contributed to weaker enrichment, possibly altered guide-target interaction. The moderate enrichment observed for *BRAF* despite its relatively lower GC content suggested that mismatch position and mismatch identity also contribute to allele discrimination.

For *PIK3CA* H1047R, the guide RNA may provide a more favorable positioning of both the somatic mutation and the intentional mismatch within the seed region relative to the PAM site, enabling strong discrimination between wild-type and mutant sequences. In contrast, the *BRAF* V596E target may have a less optimal configuration, resulting in reduced allele discrimination efficiency.

In addition, the type of nucleotide substitution may influence mismatch tolerance. The A-to-G transition in *PIK3CA* introduces a purine–purine mismatch, whereas the T-to-A transversion in *BRAF* results in a different mismatch type, which may affect EspCas9 binding and cleavage behavior. Together, these results suggest that double-mismatch sgRNA performance is influenced by several sequence-level parameters, including the position of the mutation-associated mismatch, the position of the intentional mismatch, mismatch identity, PAM-proximal seed-region context, and local sequence composition. The effectiveness of the 1-mismatch versus 2-mismatch discrimination strategy therefore appears to depend on both mismatch position and mismatch identity [33].

The consistent enrichment observed for *PIK3CA* across input levels, including low concentrations, suggests that the current guide design achieves near-optimal allele discrimination. In contrast, the lower enrichment observed for *BRAF* indicates that further optimization of guide RNA design, such as testing alternative PAM sites or mismatch positions, may improve performance.

Despite this difference, the ~15-fold enrichment achieved for *BRAF* remains meaningful for samples above 0.01% MAF. Given that *PIK3CA* H1047R is one of the most frequent driver mutations in canine mammary tumors, the strong enrichment observed for this target is particularly relevant for potential diagnostic applications [19,20,24].

### 4.3. Optimization Strategies for EspCas9-Based IVC of KRAS G12C

In our EspCas9 IVC method, *KRAS* G12C showed relatively modest enrichment. After the third IVC, the 0.1% initial mutant ratio sample increased from 0.13% to 0.29%, indicative of ~2.2-fold enrichment relative to the sgRNA-only negative control. The relatively higher enrichment of the other two targets suggested that the assessed sgRNA for *KRAS* G12C could be optimized for better performance. First, the performance might be improved if the gRNA could position the mutation optimally within the seed region. Second, as the interaction between the target sequence of *KRAS* G12C and sgRNA might be strong due to relatively high G/C ratios, an additional mismatch-engineered strategy, including a triple-mismatch design, may further improve the separation between wild-type cleavage and mutant preservation. Similar multi-mismatch CRISPR strategies have been reported to enhance single-base discrimination in mutation detection and allele-specific CRISPR applications.

### 4.4. Advantages of Sequential CRISPR IVC

Despite these target-dependent limitations, sequential CRISPR IVC still offers several important advantages over alternative enrichment approaches. The 15–160-fold enrichment achieved in this study translates to a substantial improvement in detection sensitivity for *PIK3CA* and *BRAF*. For *PIK3CA* H1047R specifically, this corresponds to an approximate 100-fold improvement relative to the typical detection limit of standard NGS. Comparison of the performance to existing technologies should be interpreted as contextual rather than as direct head-to-head benchmarking (Table 5). The other methods presented in the table, such as ddPCR, CAPP-Seq, and Safe-SeqS, are detection-oriented approaches rather than pre-analytical enrichment steps. Their reported LODs reflect standalone performance, whereas sequential CRISPR IVC functions as a pre-analytical enrichment step that showed high detection performance with conventional NGS. We anticipate that combining sequential CRISPR IVC with the aforementioned detection approaches would yield even more sensitive detection methods.

For *PIK3CA*, later-round 0% controls were not available in the original high-depth dataset because no detectable PCR product remained after the second PCR step; additional replicate values are shown separately and were not used to redefine the analytical LOD.

Despite this distinction, sequential CRISPR IVC provides unique methodological benefits, including high programmability where targeting new mutations requires only guide RNA redesign. It also offers scalability through multiplexed guide RNAs and excellent iterative potential, utilizing a semi-nested PCR design to maintain specificity across rounds, a feature lacking in methods like PNA clamps. Furthermore, the enrichment observed was strictly EspCas9-dependent; this was supported by negative controls (gRNA without EspCas9), which demonstrated no enrichment at any spike-in concentration. Wild-type-only controls showed target- and round-dependent low-level mutant-like signals, with *BRAF* and *KRAS* background values increasing across sequential rounds, while later-round *PIK3CA* 0% controls were unavailable in the original dataset because no recoverable PCR product remained after IVC. These results are consistent with low-level sequencing or amplification-derived noise rather than robust false-positive enrichment.

While detection-based methods might achieve comparable or superior LODs through direct digital counting or error correction, sequential CRISPR IVC provides the distinct advantage of physically enriching mutant templates, enabling downstream analysis by any sequencing platform without the need for specialized instrumentation. These approaches are complementary; pre-analytical enrichment via sequential CRISPR IVC could be synergistically combined with ddPCR or error-corrected NGS for even greater sensitivity gains.

### 4.5. Limitations and Future Directions

In addition to amplicon-size considerations, several limitations should be considered for clinical translation. First, the analytical LOD was established using 500 bp synthetic DNA templates rather than plasma-derived clinical cfDNA. Although these templates provided controlled spike-in materials for analytical evaluation, they do not fully reproduce the fragment-size distribution, molecular complexity, or low-input characteristics of clinical cfDNA. Second, the biological validation was performed using cell line-derived genomic DNA and conditioned-medium-derived cfDNA mimic, which may not fully represent plasma cfDNA from dogs with naturally occurring tumors. Third, *BRAF* and *KRAS* were evaluated as target-expansion experiments without independent replicate datasets, and their enrichment efficiencies remained lower than that of *PIK3CA* under the current guide designs. In addition, sequential IVC includes repeated PCR amplification between cleavage rounds, which may contribute to low-level background accumulation at very low input MAFs. We anticipate that the CRISPR IVC enrichment methods could be further refined by assessment of more targets, optimization and validation on clinical plasma cfDNA validation and molecular barcoding or UMI-based error correction.

### 4.6. Comparative Oncology Implications

This work contributes to comparative oncology that recognizes companion animals as valuable models for human cancer research [49,50]. Recent large-scale genomic studies have demonstrated that canine mammary tumors share not only the same driver genes but also identical mutational hotspots with human breast cancer—most notably *PIK3CA* H1047R, which occurs at comparable frequencies in both species [19,20]. Furthermore, systematic cross-species analysis has revealed that oncogenic hotspot mutations are broadly conserved across multiple canine cancer types, reinforcing the validity of dogs as a translational model for precision oncology [24]. The sequential CRISPR enrichment approach developed here is potentially translatable to human liquid biopsy applications, and the shared mutational landscape between canine and human cancers suggests that assays validated in veterinary settings may help inform future clinical development in human oncology.

## 5. Conclusions

We have developed a sequential CRISPR-EspCas9 enrichment strategy combined with NGS that achieves up to 160-fold mutant enrichment through iterative wild-type depletion. Within the present analytical framework, this enabled an analytical detection down to 0.001% MAF. The CRISPR enrichment could also be applied to smaller DNA with mononucleosomal size. We anticipate that the CRISPR enrichment could be further optimized for sensitive detection of low-frequency single-nucleotide mutant alleles in canine liquid biopsy.

## Figures and Tables

**Table 1:** Fisher’s exact test for LOD determination with Benjamini–Hochberg (BH) FDR correction.

Target	Input	Round	Mut/Total (Spike)	Mut/Total (Reference Control)	OR	Raw *p*	BH-adj. *p*	Significant?
*PIK3CA*	0.001%	1st	3256/7,838,455	2093/7,027,878	1.4	<10^−30^	<10^−30^	Yes
*PIK3CA*	0.001%	2nd	20,932/7,410,365	2093/7,027,878	9.5	<10^−300^	<10^−300^	Yes
*PIK3CA*	0.001%	3rd	56,210/7,471,806	2093/7,027,878	25.4	<10^−300^	<10^−300^	Yes
*BRAF*	0.01%	1st	2598/9,377,586	541/7,611,481	3.9	8.0 × 10^−235^	9.3 × 10^−235^	Yes
*BRAF*	0.01%	2nd	10,817/9,461,619	2936/8,225,328	3.2	<10^−300^	<10^−300^	Yes
*BRAF*	0.01%	3rd	18,885/7,851,119	9830/9,623,773	2.4	<10^−300^	<10^−300^	Yes
*BRAF*	0.01%	3rd vs. NC	18,885/7,851,119	438/8,626,839	47.5	<10^−300^	<10^−300^	Yes
*KRAS*	0.1%	1st	3060/1,581,846	219/1,488,437	13.2	<10^−300^	<10^−300^	Yes
*KRAS*	0.1%	2nd	3232/1,498,019	510/1,476,865	6.3	<10^−300^	<10^−300^	Yes
*KRAS*	0.1%	3rd	4850/1,697,401	1689/1,859,065	3.2	<10^−300^	<10^−300^	Yes
*KRAS*	0.1%	3rd vs. NC	4850/1,697,401	1763/1,331,035	2.2	3.6 × 10^−186^	3.6 × 10^−186^	Yes

**Note:** For *PIK3CA*, later-round 0% controls were not available because no detectable PCR product was recovered after IVC; therefore, the first-round 0% control was used as the available background reference for later-round comparisons. For *BRAF* and *KRAS*, round-matched 0% controls were used. The “3rd vs. NC” rows compare the third-round IVC sample with the matched sgRNA-only negative control and were included to support EspCas9-dependent enrichment; these comparisons were not used as the primary 0% background-based LOD criterion.

**Table 2:** Progressive enrichment of *PIK3CA* H1047R across sequential IVC rounds.

Initial Input MAF	Measured MAF in Untreated ^1^	1st IVC (%)	2nd IVC (%)	3rd IVC (%)	Fold Enrichment
1%	1.00	13.74	59.72	77	~77-fold enrichment
0.1%	0.15	1.63	11.50	23.48	~160-fold enrichment
0.01%	0.08	0.25	1.6	3.51	~41-fold enrichment
0.001%	0.05	0.04	0.28	0.75	~15-fold enrichment
0%	0.031	0.030	-	-	Below ~0.1% NGS noise level

^1^ Untreated are negative controls where the samples were incubated with only gRNA without EspCas9.

**Table 3:** Moderate enrichment of *BRAF* V596E across sequential IVC rounds.

Initial Input MAF	Measured MAF in Untreated ^1^	1st IVC (%)	2nd IVC (%)	3rd IVC (%)	Fold Enrichment
1%	0.8	2.24	10.62	13.08	~16-fold enrichment
0.1%	0.08	0.21	0.86	1.22	~15-fold enrichment
0%	0.005	0.007	0.036	0.102	Below ~0.1% NGS noise level

^1^ Untreated are negative controls where the samples were incubated with only gRNA without EspCas9.

**Table 4:** Limited enrichment performance of *KRAS* G12C across sequential IVC rounds.

Initial Input MAF	Measured MAF in Untreated ^1^	1st IVC (%)	2nd IVC (%)	3rd IVC (%)	Fold Enrichment
1%	1.4	2.24	2.29	2.32	~1.7-fold enrichment
0.1%	0.13	0.19	0.22	0.29	~2.2-fold enrichment
0%	0.014	0.015	0.035	0.091	Below ~0.1% NGS noise level

^1^ Untreated are negative controls where the samples were incubated with only gRNA without EspCas9.

**Table 5:** Contextual comparison of representative rare-mutation detection or enrichment methods summarized from representative literature.

Method	Enrichment	LOD	Programmability	Iterative Potential	Representative Reference
PNA clamps *	2–5×	~0.1%	Low	Limited	Oh et al., 2010 [37], Fouz et al., 2020 [38], Han et al., 2016 [39]
BNA-clamp PCR *	Assay-dependent	~1–5%	Low-Moderate	Limited	Nagakubo et al., 2019 [40], Tachibana et al., 2022 [41]
PNA-LNA PCR clamp	Assay-dependent	~0.01–0.1%	Low-Moderate	Limited	Watanabe et al., 2016 [42], Yoshida et al., 2017 [43], Kojima et al., 2023 [44]
COLD-PCR *	3–10×	~0.1%	Low	Moderate	Milbury et al., 2011 [45]
ddPCR*	N/A (direct detection)	~0.01%	Moderate	N/A	Watanabe et al., 2017 [46]
CAPP-Seq *	N/A (error correction)	~0.004%	High	N/A	Newman et al., 2016 [47]
Safe-SeqS *	N/A (UMI-based)	~0.01%	High	N/A	Kinde et al., 2011 [35], Fredebohm et al., 2016 [48]
DASH (Cas9 depletion) *	10–100× (depletion)	~0.1%	High	Limited	Gu et al., 2016 [28]
Single CRISPR IVC	~11×	~0.1%	High	—	This study
Sequential CRISPR IVC	~160×	~0.001%	High	Target dependent	This study

* Values represent representative literature-reported performance and are not from direct head-to-head benchmarking in this study.

## Data Availability

The raw sequencing data generated in this study have been submitted to the NCBI Sequence Read Archive (SRA) under the submission ID SUB16141057.

## Supplementary Materials

The following supporting information can be downloaded at: https://www.mdpi.com/article/10.3390/bios16060330/s1, Figure S1: Graphical illustration of mutation-site and sgRNA design for *PIK3CA* H1047R, *BRAF* V596E, and *KRAS* G12C; Figure S2: Expression and purification of EspCas9 protein; Figure S3: Semi-nested PCR strategy for sequential IVC enrichment; Figure S4: Additional NGS validation of sequential *PIK3CA* enrichment; Figure S5: Representative Sanger chromatograms and short-amplicon validation of *PIK3CA* IVC enrichment; Figure S6: Next generation sequencing (NGS) quantification of *PIK3CA* H1047R mutation across serial dilutions; Figure S7: Gel electrophoresis analysis of sequential IVC for *BRAF* and *KRAS*; Figure S8: PCR result for the *PIK3CA* 0% background control after sequential IVC; Figure S9: NGS quantification across full input range; Table S1: Design features of selected sgRNAs; Table S2: Buffer compositions for EspCas9 expression and purification; Table S3: IVC reaction conditions and DNA: EspCas9: sgRNA mass ratios; Table S4: List of primers used in this study; Table S5: Round-by-round 0% background control MAF values; Table S6: Additional *PIK3CA* NGS validation dataset.
