# Supplementary material for: Sequential CRISPR-EspCas9-Mediated Wild-Type Depletion Enhances the Detection Sensitivity of Rare Mutations for Canine Liquid Biopsy Application
Source: Biosensors (Basel). 2026 Jun 10;16(6):330. doi: 10.3390/bios16060330 (PMC13296737; doi:10.3390/bios16060330)
Supplement: Supplementary file 1 [file biosensors-16-00330-s001.zip › biosensors-4319408-supplementary.pdf]

Supplementary Material.

Cell line Development

The MGT 1 and MGT 8 cell lines used in this study were established from canine mammary tumor tissues obtained from female dogs and were provided by the laboratory of Prof. Jae-woo Hong. The original tumor specimens were collected through the Chungnam National University Veterinary Teaching Hospital. Tissue collection dates were March 23, 2023 (MGT 1) and October 11, 2023 (MGT 8). The mutation status of the *PIK3CA* H1047R target region of the cell lines were verified by Sanger sequencing. The MGT cell lines are available from the corresponding authors upon request, subject to institutional policies.

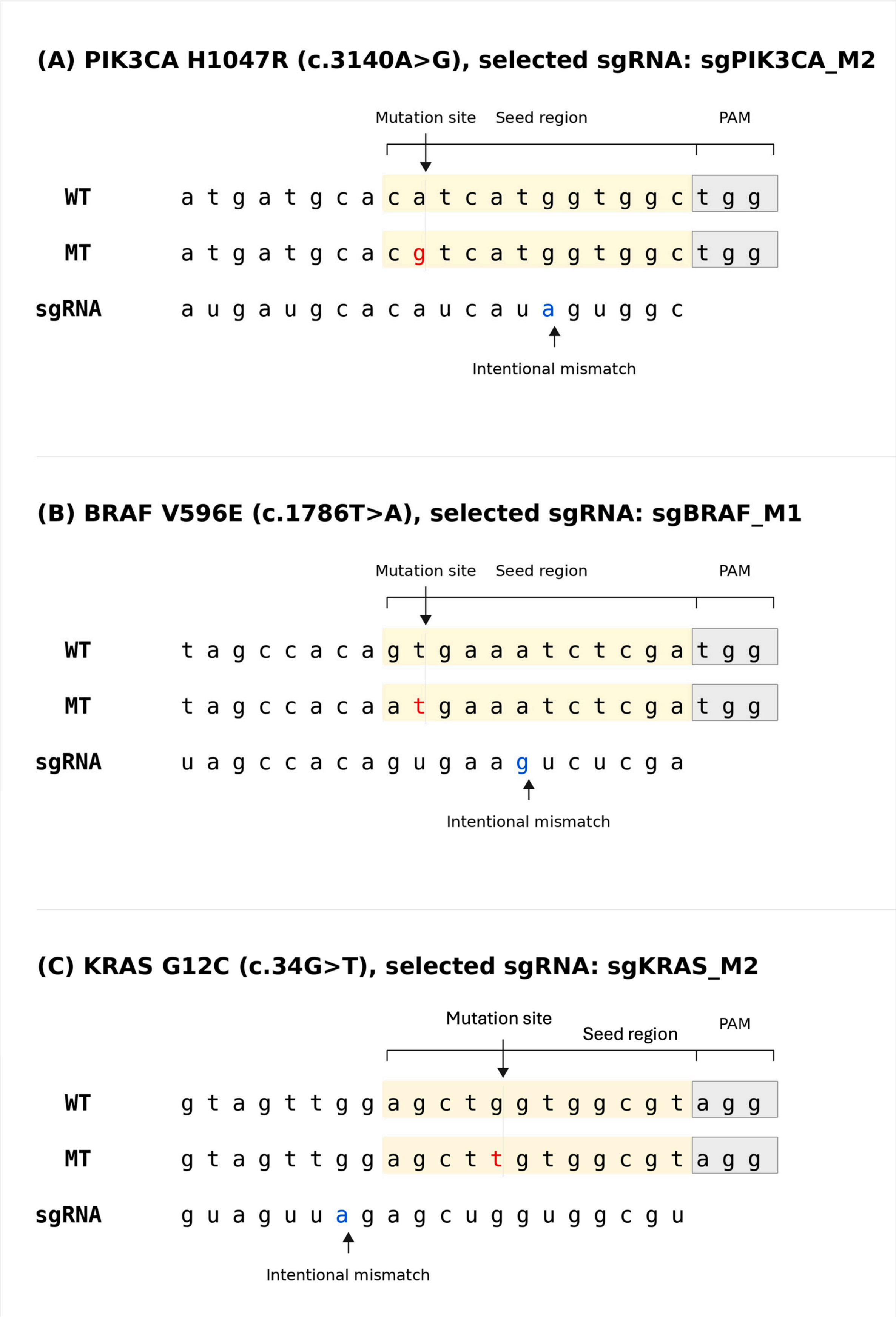

Supplementary Figure S1. Graphical illustration of mutation-site and sgRNA design for *PIK3CA* H1047R, *BRAF* V596E, and *KRAS* G12C.

Wild-type and mutant sequences are shown together with the selected sgRNA sequence for each target. The mutation-associated mismatch is marked in red, the intentional mismatch is marked in blue, the PAM sequence is indicated by a gray box, and the PAM-proximal seed region is indicated by a bracket.

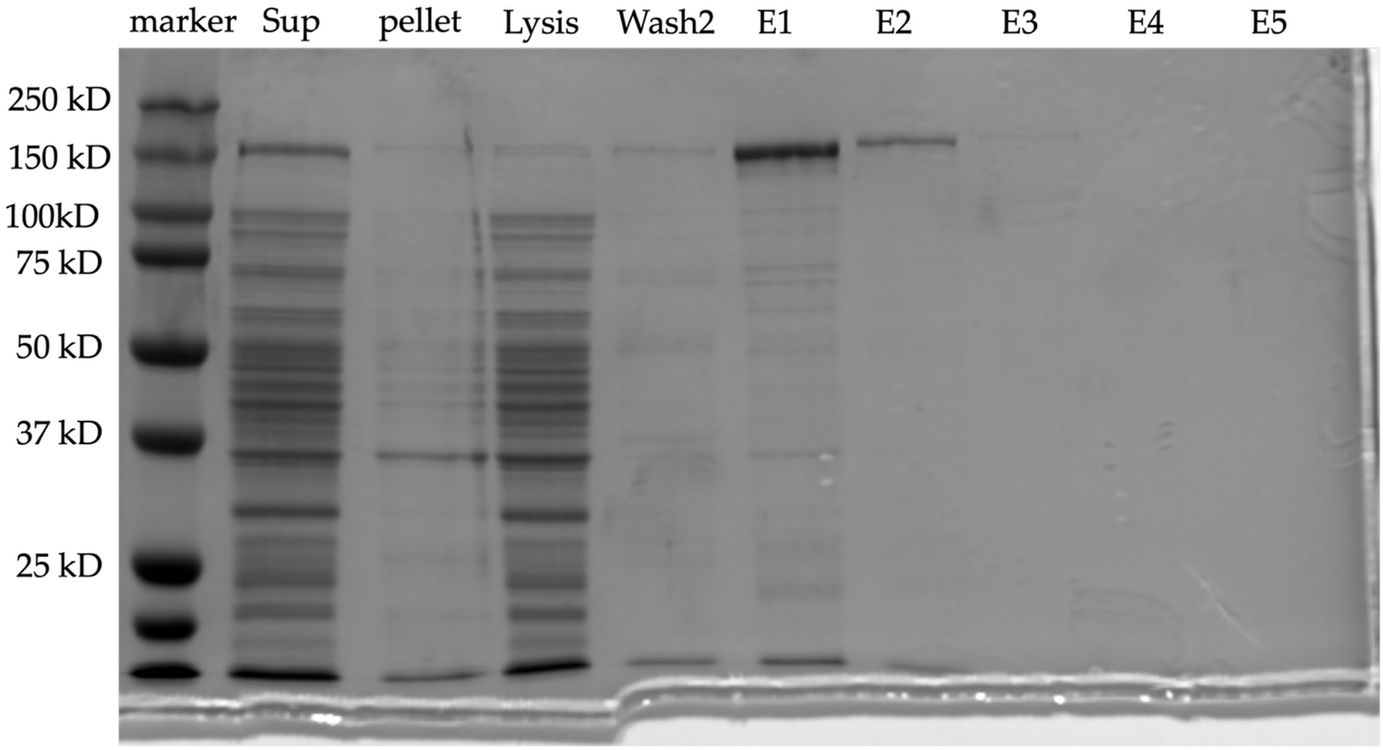

Supplementary Figure S2. Expression and purification of EspCas9 protein.

SDS-PAGE analysis of EspCas9 purification. Lane labels indicate supernatant (Sup), pellet, lysis, wash 2, and elution fractions (E1-E5). The prominent band observed at approximately 160 kDa in the elution fractions indicates successful expression and purification of the recombinant EspCas9 protein used for all *in vitro* cleavage experiments.

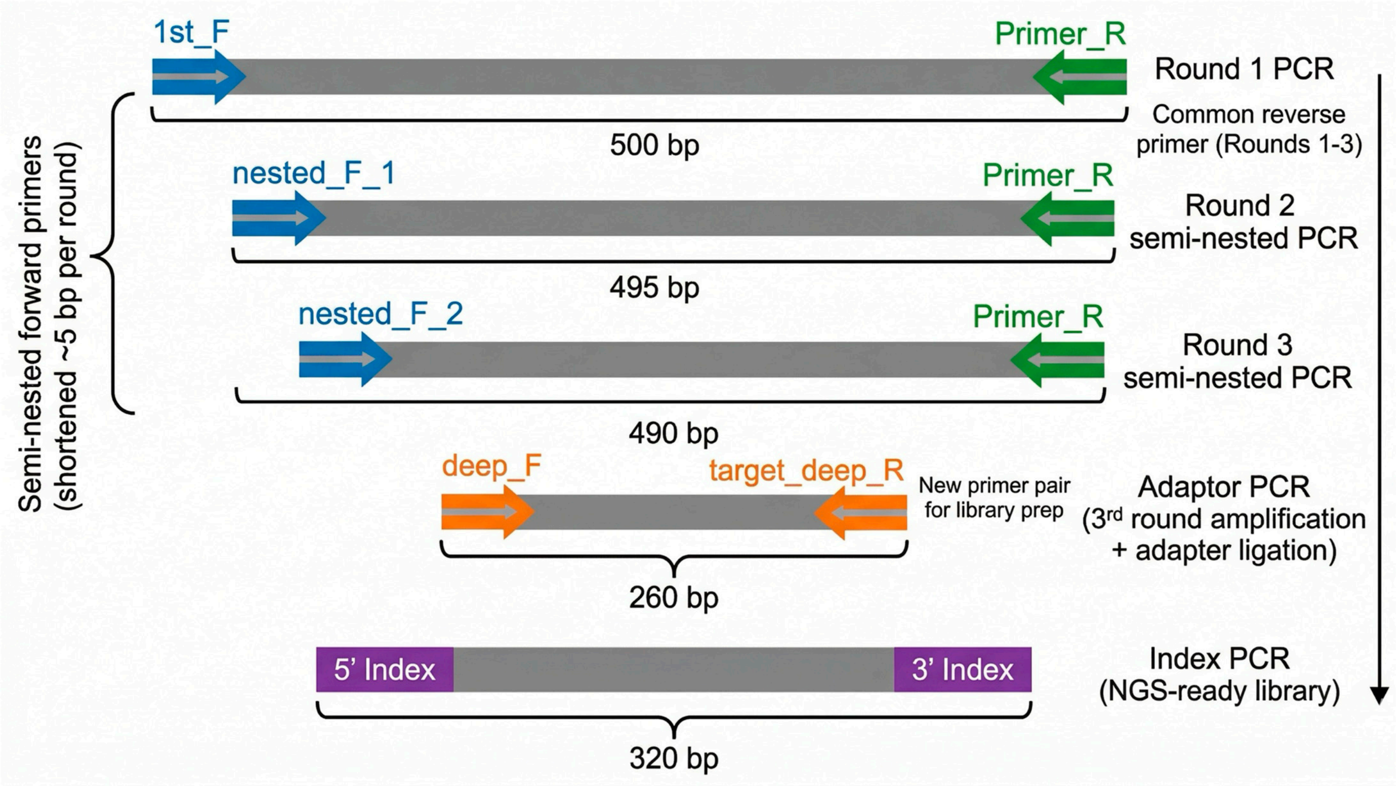

Supplementary Figure S3. Semi-nested PCR strategy for sequential IVC enrichment.

Schematic representation of the semi-nested PCR design used between sequential IVC rounds. The initial PCR generates the first target amplicon, and subsequent semi-nested PCR reactions use progressively shortened forward primers while maintaining a common reverse primer. After the third IVC round, adaptor PCR and index PCR steps are performed to generate NGS-ready libraries.

(A)

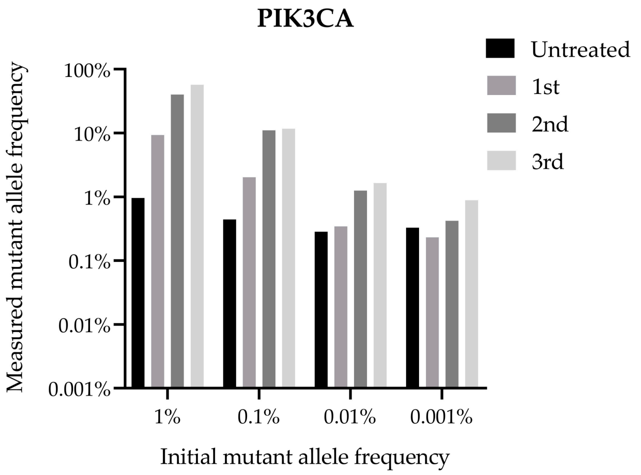

(B)

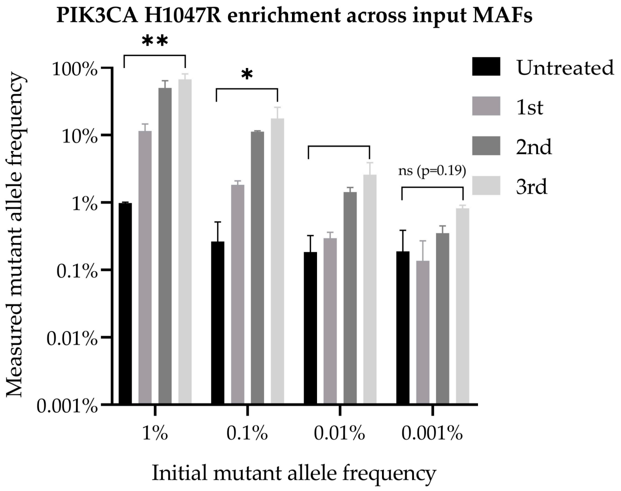

Supplementary Figure S4. Additional NGS validation of sequential *PIK3CA* enrichment.

(A) Mutant allele frequency measured in the additional *PIK3CA* NGS dataset across sgRNA-only negative control and sequential IVC rounds.
(B) Combined representation of original and additional *PIK3CA* NGS datasets for mutant-containing samples. Bars indicate mean ± SD from two independent NGS datasets. The 0% control condition was excluded from the combined graph and is shown separately in Supplementary Table 6. Statistical comparisons between the sgRNA-only negative control and third-round IVC values were performed using unpaired t-tests after log10 transformation. $^{**}p < 0.01$; $^{*}p < 0.05$, $^{\dagger}p < 0.1$; ns, not significant.

(A) (B)

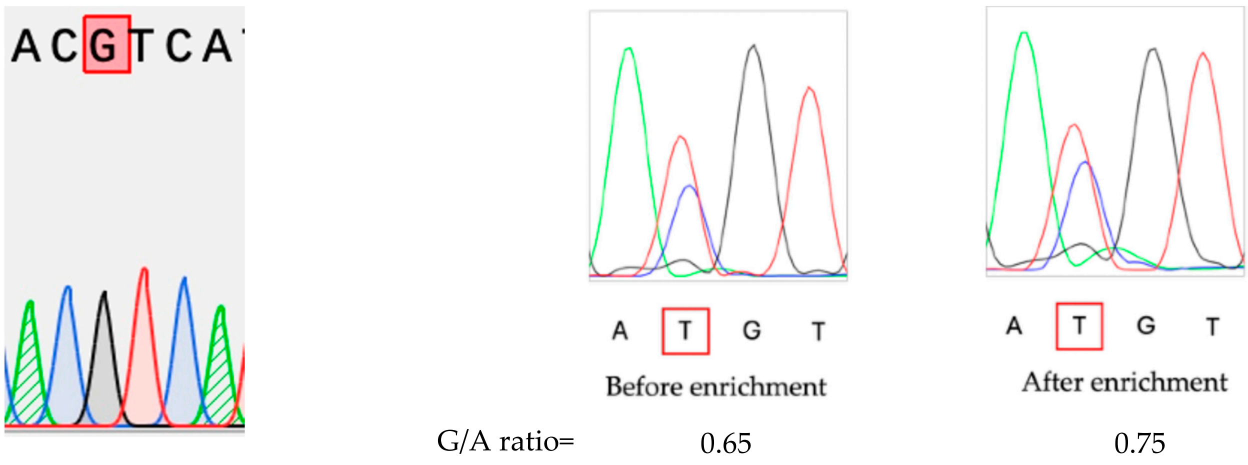

Supplementary Figure S5. Representative Sanger chromatograms and short-amplicon validation of *PIK3CA* IVC enrichment.

(A) Representative Sanger chromatogram of a PCR-amplified *PIK3CA* H1047R-positive synthetic DNA template that was not used in the enrichment experiment. This trace is provided as a chromatogram-level reference for assigning the wild-type-associated A peak and mutant-associated G peak at the *PIK3CA* H1047R mutation site.

(B) Representative Sanger chromatograms before and after one round of IVC enrichment using the 170-bp short-amplicon cfDNA mimic generated from the *PIK3CA* H1047R-positive MGT 1 cell line. The mutant-associated signal was quantified as the coding-strand-equivalent G/A peak ratio, calculated by dividing the mutant-associated G peak signal by the wild-type-associated A peak signal at the *PIK3CA* H1047R mutation site. Because the chromatograms were obtained using the reverse primer, raw C/T peak signals were converted to coding-strand-equivalent A/G signals for interpretation.

(A) (B)

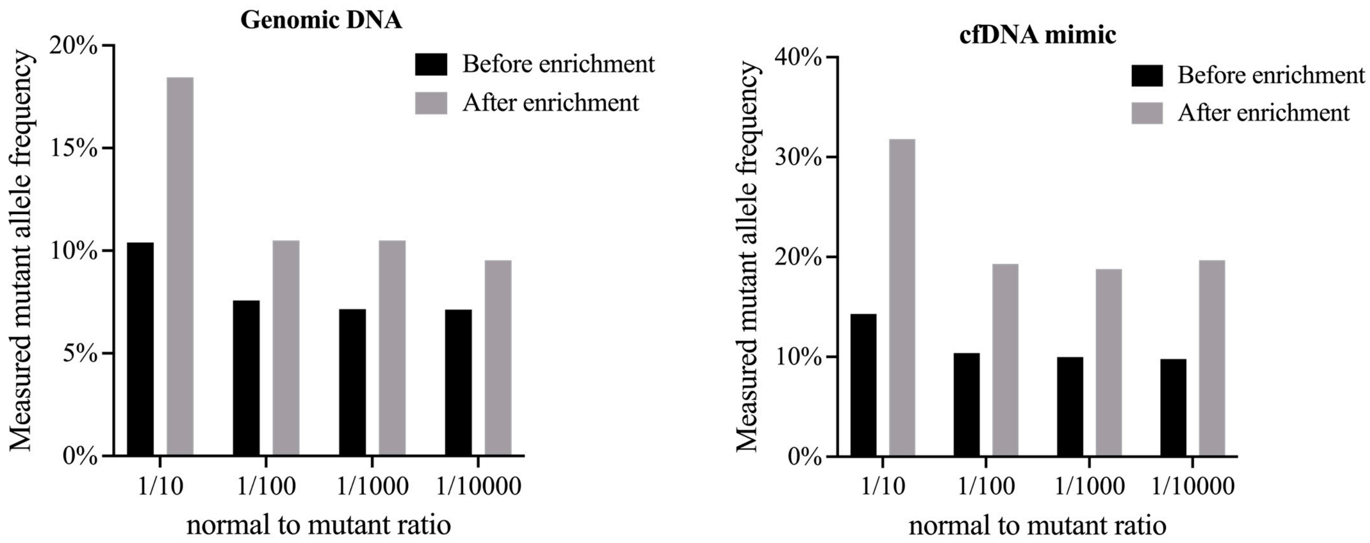

Supplementary Figure S6. Next generation sequencing (NGS) quantification of *PIK3CA* H1047R mutation across serial dilutions.

(A) gDNA and (B) cfDNA mimic samples were prepared by serial dilution of mutant DNA into wild-type DNA. NGS quantification shows an increased mutant allele frequency after IVC across the dilution series, although accurate quantification at low input levels is limited by background signal in the wild-type reference. Because the nominally wild-type reference showed measurable baseline *PIK3CA* H1047R signal, low-input dilution results should be interpreted qualitatively rather than as precise analytical LOD measurements.

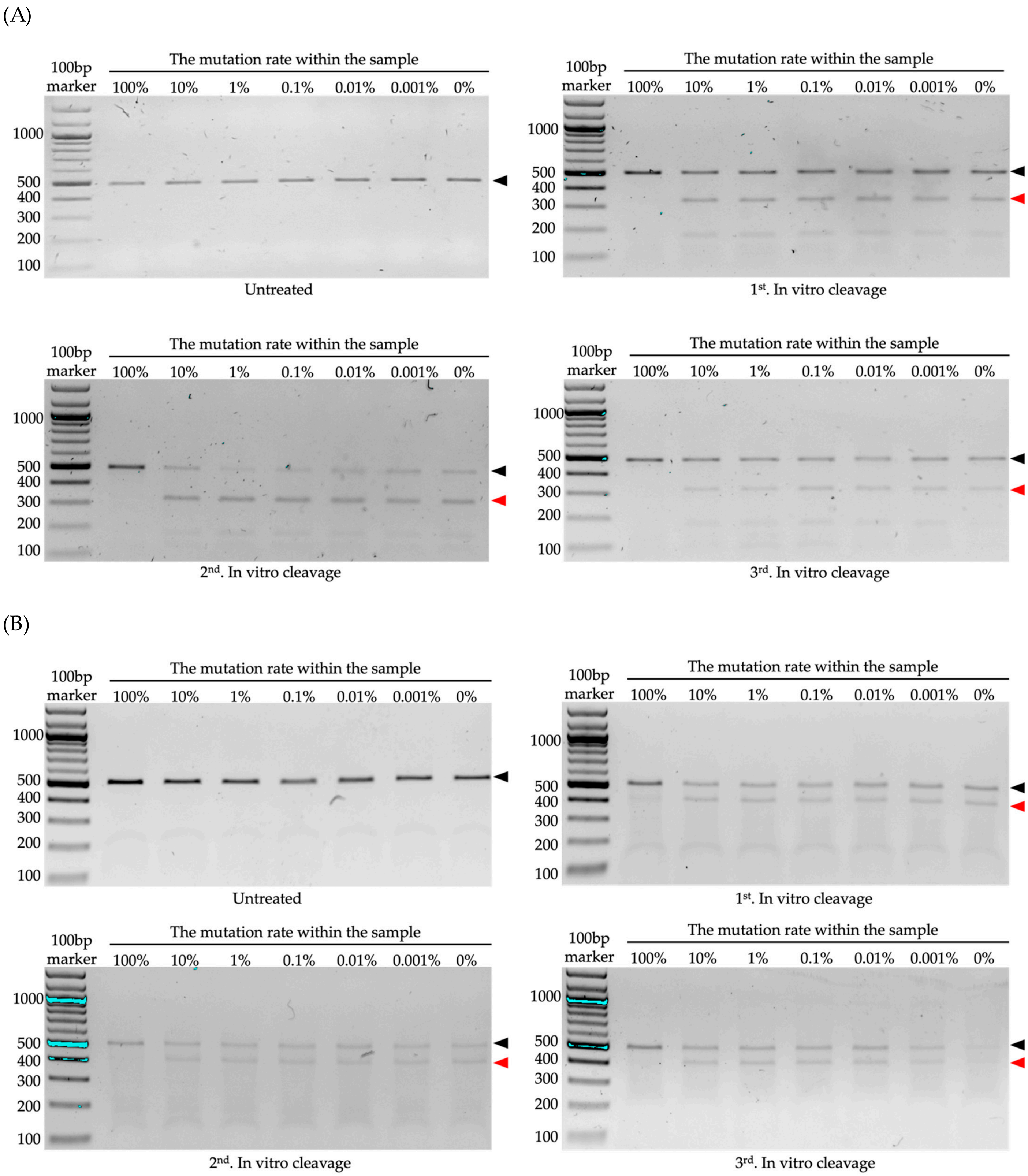

Supplementary Figure S7. Gel electrophoresis analysis of sequential IVC for *BRAF* and *KRAS*.

(A) *BRAF* and (B) *KRAS* samples were subjected to sgRNA-only negative control (named untreated) reactions without EspCas9 and sequential IVC rounds (1st – 3rd). Each lane represents different input mutant fractions. Black arrows indicate un-cleaved DNA, while red arrows indicate cleaved DNA fragments, showing the target-specific depletion effect.

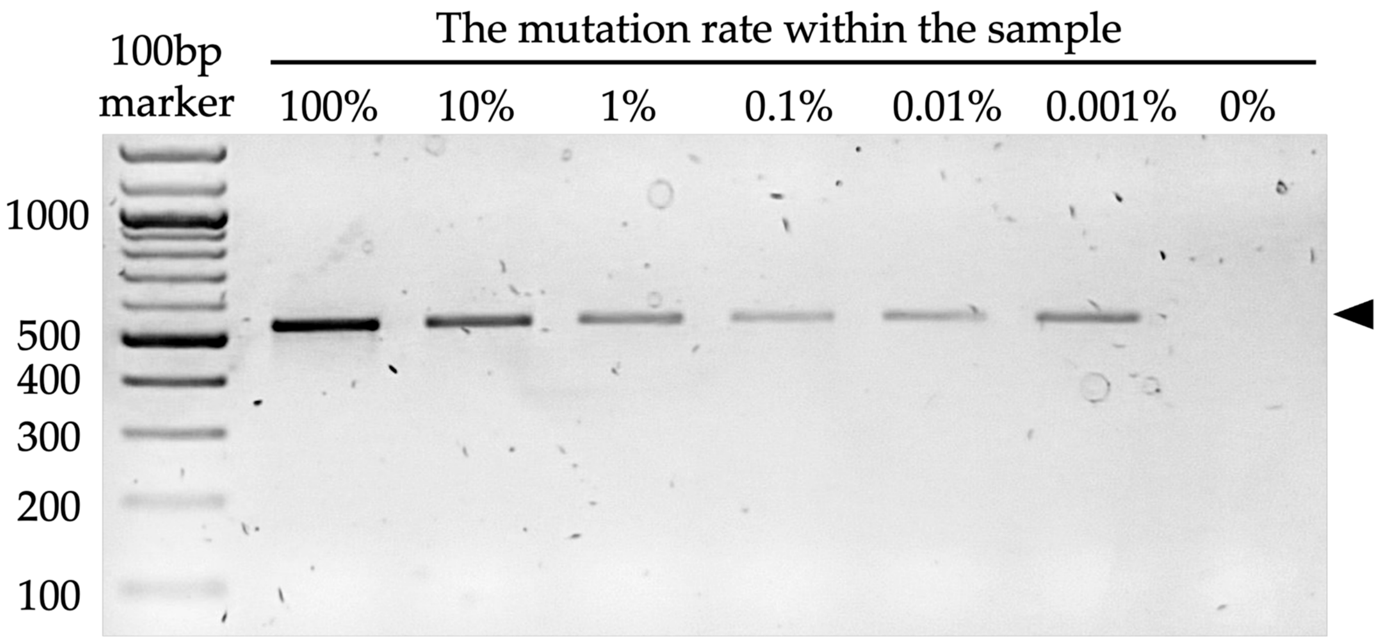

Round 2 PCR step following the first IVC round

Supplementary Figure S8. PCR result for the *PIK3CA* 0% background control after sequential IVC.

The wild-type-only *PIK3CA* background control did not yield a detectable PCR product after the second PCR step following IVC. Because no recoverable intact DNA band remained, the sample could not be purified or carried forward for later-round NGS library preparation.

(A)

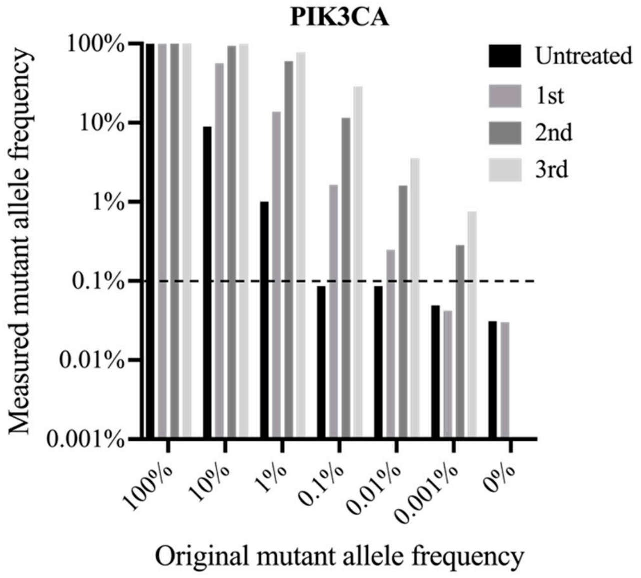

(B)

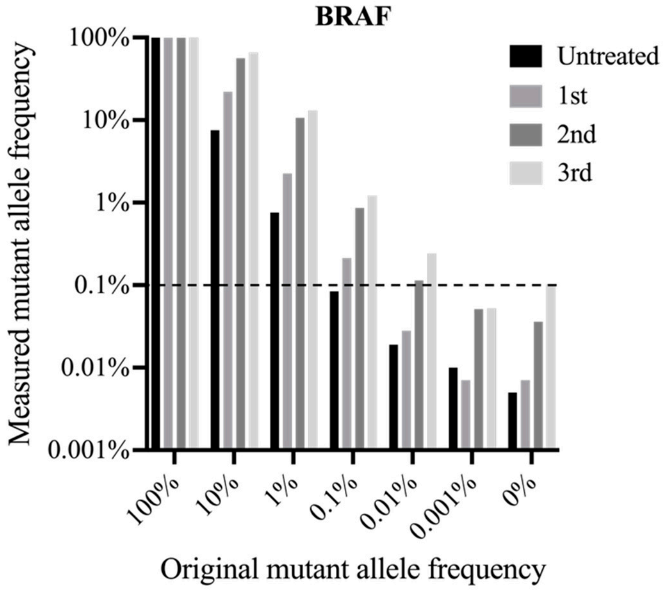

(C)

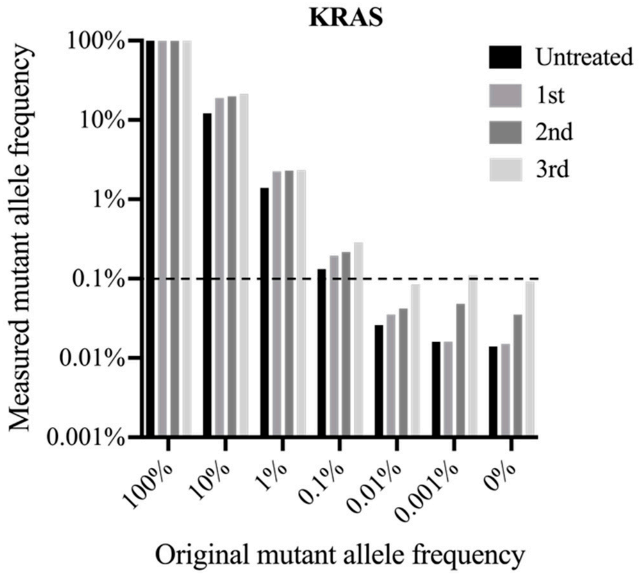

Supplementary Figure S9. NGS quantification across full input range.

(A) *PIK3CA*, (B) *BRAF* and (C) *KRAS* mutant allele frequencies were measured across the full range of input concentrations (100% to 0%) and sequential IVC rounds. The data demonstrate target-dependent enrichment efficiency and an increasing background signal at low input levels. Bars represent single NGS measurements per condition; therefore, replicate-derived error bars are not shown. The dashed line indicates the approximate background threshold of standard NGS.

Supplementary Table S1. Design features of selected sgRNAs

Target	Selected sgRNA	Mutation type	Mutation-associated mismatch position	Intentional mismatch position	Seed-region context	GC content	Enrichment outcome
PIK3CA H1047R	sgPIK3CA _M2	A > G transition	Within PAM-proximal seed region	Within PAM-proximal seed region	Both mismatch sites positioned in the seed region	50%	Strong enrichment
BRAF V596E	sgBRAF _M1	T > A transversion	Within PAM-proximal seed region	Within PAM-proximal seed region	Both mismatch sites positioned in the seed region	45%	Moderate enrichment
KRAS G12C	sgKRAS _M2	G > T transversion	Within seed region	More distal from PAM	Less favorable mismatch configuration	60%	Limited enrichment

Supplementary Table S2. Buffer compositions for EspCas9 expression and purification

Buffer	Reagent
Lysis buffer (= Wash 1 buffer)	50mM HEPES pH 7.5, 500mM NaCl, 5mM imidazole
Wash 2 buffer	50mM HEPES pH 7.5, 500mM NaCl, 30mM imidazole
Elution	50mM HEPES pH 7.5, 500mM NaCl, 250mM imidazole
Storage	50mM HEPES pH 7.5, 500mM NaCl, 1mM DTT, 10% glycerol

Supplementary Table S3. IVC reaction conditions and DNA: EspCas9: sgRNA mass ratios.

Target	Reaction volume (μL)	DNA template (ng)	EspCas9 (μg)	sgRNA (ng)	Approx. EspCas9 final concentration	Approx. sgRNA final concentration	DNA: EspCas9: sgRNA mass ratio	Approx. EspCas9: sgRNA molar ratio
PIK3CA H1047R	30 μL	30 ng	1.22 μg	100 ng	~254 nM	~ 105 nM	1: 40.7: 3.3	~ 2.4:1
BRAF V596E	30 μL	30 ng	1.22 μg	300 ng	~254 nM	~ 315 nM	1: 40.7: 10.0	~ 0.8:1
KRAS G12C	30 μL	30 ng	1.22 μg	100 ng	~254 nM	~ 105 nM	1: 40.7: 3.3	~ 2.4:1

Supplementary Table S4. List of primers used in this study

	Sequence (5′ – 3′)
1st_F	GTG GGG TAA AGA GGA CAG AAA G
Nested_F_1	GGT AAA GAG GAC AGA AAG TGT TG
Nested_F_2	AGA GGA CAG AAA GTG TTG ATA AGG
Primer_R	CAT GGA TTG TGC AAT TCC TAT GC
Deep_F	ACACTCTTTCCCTACACGACGCTCTTCCGATCTTCAATGATGCTTGGCTCTGGA
PIK3CA_deep_short_F	ACACTCTTTCCCTACACGACGCTCTTCCGATCTGAACAAGAGGCTTTGGAATAT
PIK3CA_deep_R	GTGACTGGAGTTCAGACGTGTGCTCTTCCGATCTCAGTTCAAAGCATGCTGCTTAA
BRAF_deep_R	GTGACTGGAGTTCAGACGTGTGCTCTTCCGATCTAGTAGCACCTCAGGGTCCAA
KRAS_deep_R	GTGACTGGAGTTCAGACGTGTGCTCTTCCGATCTGAATTAGCTGTATCGTCAAGGC

Supplementary Table S5. Round-by-round 0% background control MAF values

Target	Round	Mutant-like reads	Total reads	0% control MAF (%)
PIK3CA H1047R	sgRNA-only NC	2,363	7,587,932	0.031
PIK3CA H1047R	1st IVC	2,093	7,027,878	0.030
PIK3CA H1047R	2nd IVC	-	-	-
PIK3CA H1047R	3rd IVC	-	-	-
BRAF V596E	sgRNA-only NC	438	8,626,839	0.005
BRAF V596E	1st IVC	541	7,611,481	0.007
BRAF V596E	2nd IVC	2,936	8,225,328	0.036
BRAF V596E	3rd IVC	9,830	9,623,773	0.102
KRAS G12C	sgRNA-only NC	219	1,549,646	0.0141
KRAS G12C	1st IVC	219	1,488,437	0.015
KRAS G12C	2nd IVC	510	1,476,865	0.035
KRAS G12C	3rd IVC	1,689	1,859,065	0.091

For *PIK3CA*, later-round 0% controls were not available because no detectable PCR product was recovered for subsequent second- and third-round processing after IVC. For *BRAF* and *KRAS*, residual PCR products were recovered and used to estimate round-matched 0% background MAF values.

Supplementary Table S6. Additional *PIK3CA* NGS validation dataset

Target	Input	sgRNA-only NC	1st IVC	2nd IVC	3rd IVC
PIK3CA H1047R	1%	0.958%	9.318%	40.039%	57.332%
PIK3CA H1047R	0.1%	0.441%	2.020%	11.008%	11.704%
PIK3CA H1047R	0.01%	0.283%	0.343%	1.251%	1.641%
PIK3CA H1047R	0.001%	0.328%	0.231%	0.422%	0.883%
PIK3CA H1047R	0%	0.171%	0.239%	0.355%	0.534%

Note: The 0% *PIK3CA* values from the additional lower-depth validation dataset are shown for transparency but were not included in the combined graph, statistical comparisons, or analytical LOD interpretation because matched later-round 0% controls were not available across both original and additional datasets.
